# Dysregulated iron homeostasis in dystrophin-deficient cardiomyocytes: correction by gene editing and pharmacological treatment

**DOI:** 10.1093/cvr/cvad182

**Published:** 2023-12-12

**Authors:** Kalina Andrysiak, Gabriela Machaj, Dominik Priesmann, Olga Woźnicka, Alicja Martyniak, Guillem Ylla, Marcus Krüger, Elżbieta Pyza, Anna Potulska-Chromik, Anna Kostera-Pruszczyk, Agnieszka Łoboda, Jacek Stępniewski, Józef Dulak

**Affiliations:** Department of Medical Biotechnology, Faculty of Biochemistry, Biophysics and Biotechnology, Jagiellonian University, Gronostajowa 7, 30-387 Kraków, Poland; Laboratory of Bioinformatics and Genome Biology, Faculty of Biochemistry, Biophysics and Biotechnology, Jagiellonian University, Kraków, Poland; Institute for Genetics, Cologne Excellence Cluster on Cellular Stress Responses in Aging-Associated Diseases, University of Cologne, Cologne, Germany; Department of Cell Biology and Imaging, Institute of Zoology and Biomedical Research, Faculty of Biology, Jagiellonian University, Kraków, Poland; Department of Medical Biotechnology, Faculty of Biochemistry, Biophysics and Biotechnology, Jagiellonian University, Gronostajowa 7, 30-387 Kraków, Poland; Laboratory of Bioinformatics and Genome Biology, Faculty of Biochemistry, Biophysics and Biotechnology, Jagiellonian University, Kraków, Poland; Institute for Genetics, Cologne Excellence Cluster on Cellular Stress Responses in Aging-Associated Diseases, University of Cologne, Cologne, Germany; Department of Cell Biology and Imaging, Institute of Zoology and Biomedical Research, Faculty of Biology, Jagiellonian University, Kraków, Poland; Department of Neurology, Medical University of Warsaw, Warsaw, Poland; Department of Neurology, Medical University of Warsaw, Warsaw, Poland; Department of Medical Biotechnology, Faculty of Biochemistry, Biophysics and Biotechnology, Jagiellonian University, Gronostajowa 7, 30-387 Kraków, Poland; Department of Medical Biotechnology, Faculty of Biochemistry, Biophysics and Biotechnology, Jagiellonian University, Gronostajowa 7, 30-387 Kraków, Poland; Department of Medical Biotechnology, Faculty of Biochemistry, Biophysics and Biotechnology, Jagiellonian University, Gronostajowa 7, 30-387 Kraków, Poland

**Keywords:** DMD, Induced pluripotent stem cells, hiPSC-CM, Iron overload, Cardiomyopathy, MitoNEET, CISD1, Cardiomyocytes, Duchenne muscular dystrophy, Deferoxamine, Pioglitazone, CRISPR/Cas9

## Abstract

**Aims:**

Duchenne muscular dystrophy (DMD)-associated cardiomyopathy is a serious life-threatening complication, the mechanisms of which have not been fully established, and therefore no effective treatment is currently available. The purpose of the study was to identify new molecular signatures of the cardiomyopathy development in DMD.

**Methods and results:**

For modelling of DMD-associated cardiomyopathy, we prepared three pairs of isogenic control and dystrophin-deficient human induced pluripotent stem cell (hiPSC) lines. Two isogenic hiPSC lines were obtained by CRISPR/Cas9-mediated deletion of *DMD* exon 50 in unaffected cells generated from healthy donor and then differentiated into cardiomyocytes (hiPSC-CM). The latter were subjected to global transcriptomic and proteomic analyses followed by more in-depth investigation of selected pathway and pharmacological modulation of observed defects. Proteomic analysis indicated a decrease in the level of mitoNEET protein in dystrophin-deficient hiPSC-CM, suggesting alteration in iron metabolism. Further experiments demonstrated increased labile iron pool both in the cytoplasm and mitochondria, a decrease in ferroportin level and an increase in both ferritin and transferrin receptor in DMD hiPSC-CM. Importantly, CRISPR/Cas9-mediated correction of the mutation in the patient-derived hiPSC reversed the observed changes in iron metabolism and restored normal iron levels in cardiomyocytes. Moreover, treatment of DMD hiPSC-CM with deferoxamine (DFO, iron chelator) or pioglitazone (mitoNEET stabilizing compound) decreased the level of reactive oxygen species in DMD hiPSC-CM.

**Conclusion:**

To our knowledge, this study demonstrated for the first time impaired iron metabolism in human DMD cardiomyocytes, and potential reversal of this effect by correction of *DMD* mutation or pharmacological treatment. This implies that iron overload-regulating compounds may serve as novel therapeutic agents in DMD-associated cardiomyopathy.

## Graphical Abstract

**Graphical Abstract:**
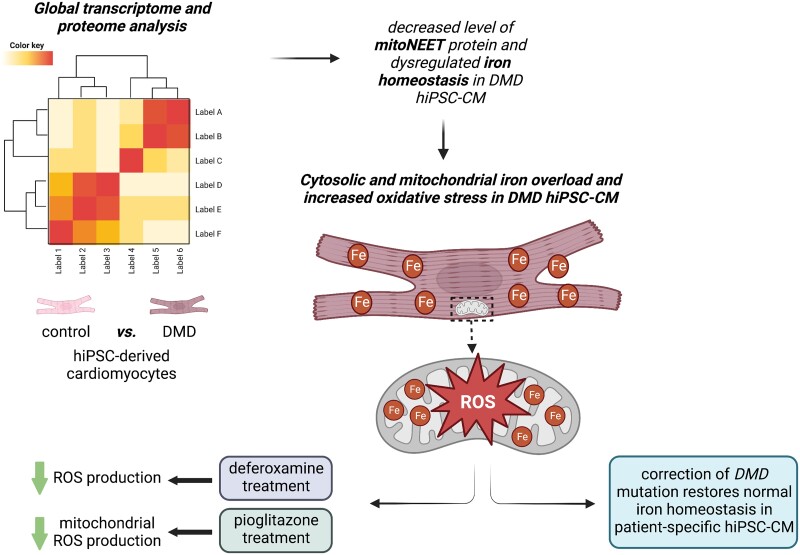

**Time of primary review: 38 days**

## 1. Introduction

Duchenne muscular dystrophy (DMD) is a rare X-linked genetic disorder that occurs as a result of mutations in the *DMD* gene encoding dystrophin, a protein found mainly in muscle cells as a component of the dystrophin–associated glycoprotein complex. It provides a link between the cell sarcolemma and the cytoskeleton, and its function is mostly associated with maintaining the structural integrity of the cell membrane. Thus, since muscle cells are continuously subjected to mechanical stimulation, the lack of dystrophin leads to their damage^1^ with clinical symptoms primarily related to progressive muscle weakness.

In the later stages of disease progression, the respiratory muscles and heart are also affected, leading to breathing problems and cardiomyopathy development. Until recently, the respiratory failure was the main cause of death in DMD patients before reaching the age of twenty,^2^ however, progress in patient care, wide use of respiratory devices, and constant medical monitoring of cardiomyopathy development at least partially solved this problem and extended the lifetime.^3^

Nevertheless, cardiomyopathy arising in patients with DMD is a life-threatening complication and it is currently considered to be the leading cause of death in DMD patients. Its clinical symptoms are observed relatively late in disease progression—in adult patients over 20–30 years old, however, some initial signs of cardiac deterioration, without the clinical outcome yet, are detected much earlier, most often through abnormal signals during ECG.^4^ So far, no effective treatment for DMD-associated cardiomyopathy has been developed. Accordingly, better understanding of molecular dysfunction of DMD cardiomyocytes may offer the chance for therapeutic improvements.

Human induced pluripotent stem cell (hiPSC) technology combined with the Clustered Regularly Interspaced Short Palindromic Repeats (CRISPR)/CRISPR-associated protein 9 (Cas9) (CRISPR/Cas9) gene editing system provides a powerful methodology for *in vitro* studies of genetic diseases. It is particularly beneficial for cardiac disorders, as their modelling in research is challenging due to the virtually impossible acquisition of patient-specific primary cells from a biopsy and an imperfect representation of disease in available animal models. In the present study, we utilized the *in vitro* model based on dystrophin-deficient hiPSC-derived cardiomyocytes (hiPSC-CM) and respective isogenic controls to identify the molecular background of DMD-associated cardiomyopathy using global proteomic and transcriptomic analyses followed by validation of selected altered pathways highlighted in the obtained omics data. One of the identified targets was mitoNEET, an outer membrane mitochondrial protein involved in the maintenance of energy homeostasis, lipid metabolism, and regulation of labile iron level in mitochondria.^5^ The protein was described in 2004 as a target for pioglitazone, a drug used for the treatment of type II diabetes, which stabilizes the mitoNEET protein.^6^ As the previous studies on DMD demonstrated increased reactive oxygen species (ROS) production and altered level of genes and proteins involved in iron metabolism^7,8^ and the mitoNEET protein, indicated in our data, regulates the transport of iron from mitochondria to cytoplasm, we made a hypothesis, that disturbance in iron level might underlie the pathogenesis of DMD-associated cardiomyopathy. Our results indicated iron overload in the cytoplasm and mitochondria of DMD hiPSC-CM, increased ROS production, and disturbed level of proteins involved in the regulation of iron homeostasis. Importantly, CRISPR/Cas9-mediated correction of the mutation in the patient-derived hiPSC reversed the observed changes in iron metabolism and restored normal iron levels in cardiomyocytes. Furthermore, treatment of DMD hiPSC-CM with deferoxamine (DFO, iron chelator) or pioglitazone decreased the level of ROS in DMD hiPSC-CM.

## 2. Methods

### 2.1 hiPSC culture

**Figure 1:**
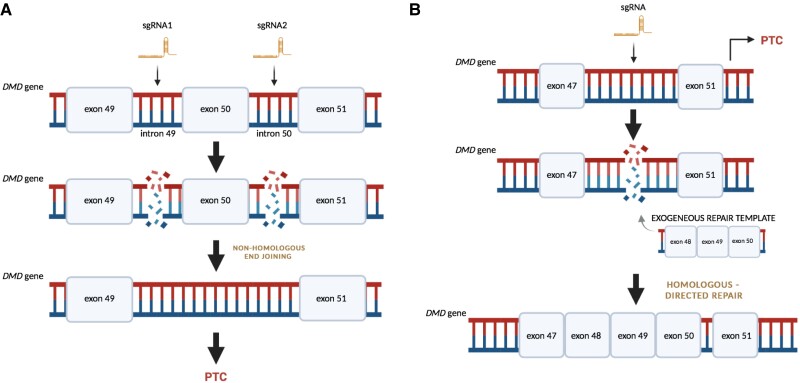
Scheme of CRISPR/Cas9-mediated introduction or correction of the DMD mutation in hiPSC. (*A*) An example of non-homologous end joining (NHEJ)-mediated introduction of an exon 50 deletion in the DMD gene in the control hiPSC (DMB01 and DMB02) and (*B*) Homology-directed repair (HDR) double strand breaks (DSB) repair mechanism of deletion of exons 48–50 in DMD patient-derived hiPSC (DMB03) used in this study. PTC, premature termination codon. Created with BioRender.com.

The generation, culture, characterization, and cardiac differentiation of hiPSC (see Supplementary material online, *Table S1*) was performed as previously reported^9^ and described in detail in the Supplementary Data. CRISPR/Cas9-mediated deletion of *DMD* exon 50 in the control hiPSC line, as well as repair of *DMD* mutation in patient-derived cells, was performed as outlined in the Supplementary Data (and *Figure 1*). Quantitative PCR and western blot analyses were performed as outlined in the Supplementary Data. The hiPSC lines used in this study were generated from the blood samples collected from two donors upon obtaining an informed consent in accordance with the Declaration of Helsinki and with the approval of the Institutional Review Board and the Bioethical Committee (number of approval: 122.6120.303.2016 and KB/111/2019).

### 2.2 Transcriptomic and proteomic analyses

For transcriptomic and proteomic analyses, control and DMD hiPSC-CM samples were prepared from two separate differentiation runs of two isogenic hiPSC lines obtained from different donors (DMB01-CTRL and DMD and DMB02-CTRL and DMD).

RNA libraries were prepared using the Ion AmpliSeq™ Transcriptome Human Gene Expression Kit. The libraries were then combined in equimolar amounts and sequenced on the Ion Proton™ Sequencer using the Ion PI™ Hi-Q™ Sequencing 200 Kit and the Ion PI™ Chip Kit v3 (all from Thermo Fisher Scientific). Obtained datasets were deposited in public repository (GEO repository: GSE226170).

Proteomic analysis was performed on a Q-Exactive Plus instrument coupled to an EASY nLC 1000 UHPLC system (both from Thermo Fisher Scientific). Full MS scans were performed at a resolution of 70,000, an automated gain control (AGC) target of 3e^6^ and a maximum injection time (IT) of 20 ms. The Top10 most abundant precursors were selected for fragmentation scans as a resolution of 17,500, an AGC target of 5e^5^ and a maximum IT of 60 ms. Obtained datasets were deposited in public repositiory (RODBUK Cracow Open Research Data Repository and are available at the following link: https://doi.org/10.57903/UJ/Y6JNUZ).

A protocol of the sample preparation and detailed methodology are described in the Supplementary Data.

### 2.3 ROS and mitochondrial ROS level determination

For the ROS level determination, 150 000 hiPSC-CM were seeded onto a Geltrex™-coated 24-well plate. After 48 h, cells were harvested and incubated with CellROX™ Deep Red Reagent (Thermo Fisher Scientific) (for total cellular ROS level determination) or mitoSOX™ Red probe (Thermo Fisher Scientific) (for mitochondrial ROS level determination) according to the manufacturer’s instructions. Briefly, the reagent was diluted in culture medium to a concentration of 5 µM and added to the cells for 30 or 10 min incubation at 37°C in the darkness, respectively. After that, the cells were washed with PBS and analysed using the LSR Fortessa flow cytometer.

### 2.4 Labile iron level determination

For labile iron level determination 150 000 hiPSC-CM were seeded onto a Geltrex™-coated 24-well plate. After three days, cells were washed with PBS and incubated with 5 µM Calcein-AM (Sigma-Aldrich) diluted in PBS for 30 min at 37°C in the darkness. Subsequently, the cells were washed with PBS, detached and centrifuged at 200 × *g* for 5 min. The pellet was reconstituted in PBS with 0.2 µg/mL DAPI and the degree of decrease in the fluorescent signal corresponding to iron-mediated quenching was analysed using the LSR Fortessa flow cytometer.

### 2.5 Mitochondrial iron level determination

For the mitochondrial iron level determination, 150 000 hiPSC-CM were seeded onto a Geltrex™-coated 24-well plate. After 48 h, cells were harvested and incubated with 5 µM Rhodamine B-[(1,10-phenanthroline-5-yl)-aminocarbonyl]benzyl ester (RPA) reagent (Squarix) diluted in Hanks’ Balanced Salt Solution (HBSS) (Thermo Fisher Scientific) for 15 min at 37°C in the darkness. Subsequently, the cells were washed with HBSS, centrifuged (200 × *g*, 5 min, 4°C) and resuspended in HBSS. Following the next 15 min of incubation at 37°C in darkness, cells were centrifuged and resuspended in fresh HBSS. The degree of decrease in fluorescent signal corresponding to mitochondrial iron-mediated quenching was analysed using the LSR Fortessa flow cytometer.

### 2.6 Transmission electron microscopy (TEM) imaging

For transmission electron microscopy (TEM) imaging, hiPSC-CM were detached from the surface using TrypLE™ Select Enzyme and centrifuged at 200 × *g* for 5 min. Pellet of cells was fixed with 2.5% glutaraldehyde (Sigma-Aldrich) in 0.1 M cacodylate buffer (Sigma-Aldrich) overnight at 4°C, followed by 1% osmium tetroxide for 1 h at RTemp. Subsequently, the hiPSC-CM pellet samples were dehydrated in series of graded ethanol (EtOH) (10 min in 50% EtOH, 10 min in 70% EtOH, 10 min in 96% EtOH, 2 × 15 min in 100% EtOH), followed by incubation in propylene oxide (2 × 5 min) and embedding in Poly/Bed® 812 (Polysciences) epoxy formulation at 68°C. In the next step, ultrathin sections (∼65 nm) were cut using a microtome, placed on 300-mesh Formvar/Carbon supported Copper grids and contrasted using uranyl acetate and lead citrate. TEM imaging was performed using the JEM-2100 HT electron microscope (JEOL, Japan) at an accelerating voltage of 80 kV.

### 2.7 Statistical analysis

Statistical significance of qPCR, cytometric measurements, and microelectrode array (MEA) data was assessed using GraphPad Prism version 9.3.1 software (San Diego, CA, USA, www.graphpad.com) by the unpaired Student’s *t*-test for the comparison of two datasets with normal distribution and two-way ANOVA with Tukey’s correction for multiple comparisons for more datasets. All data are presented as mean ± S.E.M. *N* describes the number of samples coming from independent hiPSC-CM lines, while *n* represents the number of samples coming from separate differentiation runs of each cell line. Data with *P* < 0.05 were considered statistically significant.

For transcriptome data, the obtained results of gene expression were normalized and differential analysis was performed with the DESeq2 package available in the R software version 3.3.3. In case of proteome data statistical analysis was performed within Perseus (1.5.5.3). Potential contaminants and reverse peptides were excluded and intensities were log2-transformed. Two-sided *t*-tests were performed to identify differentially expressed proteins between experimental conditions.

All other methods including western blot, reverse transcription, quantitative PCR, immunofluorescent staining, MEA, and reagents preparation are described in detail in the Supplementary Data.

## 3. Results

### 3.1 The proteomics and transcriptomics signatures of DMD

**Figure 2:**
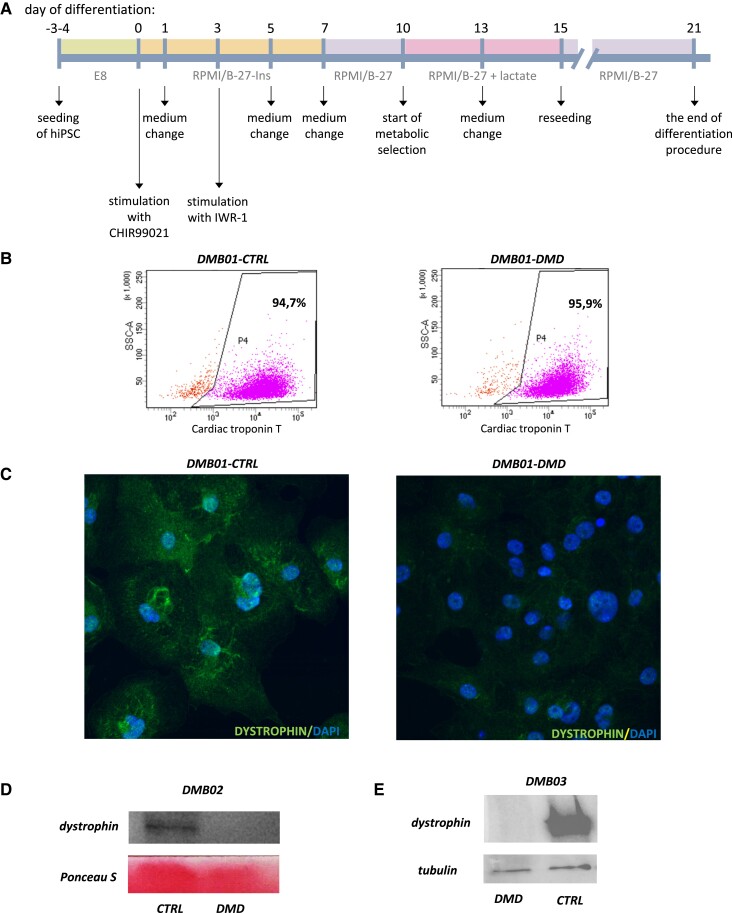
Differentiation of hiPSC to cardiomyocytes and confirmation of CRISPR/Cas9-mediated introduction and repair of the mutation in the DMD gene at the protein level. (*A*) Scheme of cardiac differentiation based on Wnt/β-catenin pathway modulation; (*B*) representative dot plots of flow cytometric analysis of cardiac differentiation efficiency in DMB01 cells calculated as percentage of troponin T-positive cells (shown as events collected in P4 gate); (*C*) representative pictures of DMB01-CTRL and DMB01-DMD hiPSC-CM stained for dystrophin, scale bars represent 50 µm; (*D*) western blot analysis of dystrophin in control and DMD DMB02 hiPSC-CM shown as representative pictures (*n* = 2, separate differentiation runs); (*E*) western blot analysis of dystrophin in DMD and corrected (CTRL) hiPSC-CM shown as representative pictures (*n* = 2, separate differentiation runs).

To unveil the molecular effects of dystrophin deficiency in hiPSC-CM, we took a proteomic and transcriptomic approach. In addition to the previously established pair of isogenic control and dystrophin-deficient hiPSC (DMB01),^10^ we applied CRISPR/Cas9 gene editing to delete *DMD* exon 50 from the other control DMB02 hiPSC line (*Figure 1* and Supplementary material online, *Figure S1*) and confirmed the pluripotent phenotype and normal karyotype of these cells (see Supplementary material online, *Figure S2*). We then subjected both cell lines to cardiac differentiation using small molecule-mediated regulation of the WNT pathway (*Figure 2A*). Flow cytometric analysis of cardiac troponin T expression (*Figure 2B*) revealed high efficiency of hiPSC-CM generation while immunocytochemical (*Figure 2C*) and western blot analysis (*Figure 2D*—the result for DMB02 hiPSC-CM, the result for DMB01 presented elsewhere^10^) confirmed lack of dystrophin in obtained cells. Eventually, global proteomic and transcriptomic analyses were performed, using material from isogenic control and DMD hiPSC-CM lines from two separate differentiation runs (marked as CTRL1.1 and CTRL1.2 for DMB01-CTRL samples and DMD1.1 and DMD1.2, for DMB01-DMD samples, a similar description applies to DMB02 lines).

**Figure 3:**
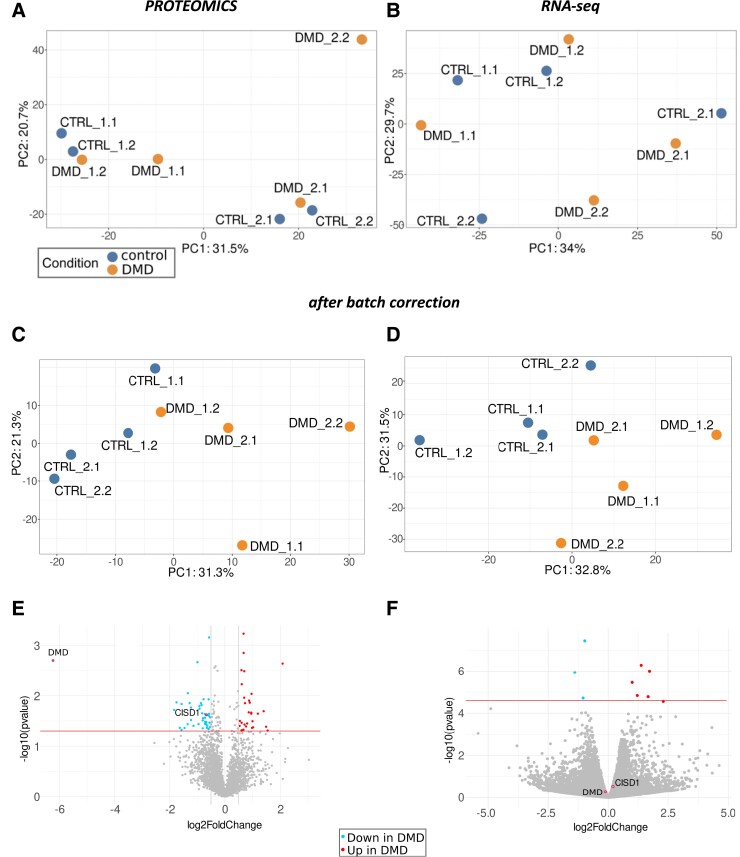
Transcriptomic and proteomic effect of DMD vs. control hiPSC-CM. The principal component analysis (PCA) of the (*A*) proteomic and (*B*) transcriptomic samples, based on log2-counts and vst-normalized counts respectively, shows that samples group together based on donor and batch. After correcting the donor and batch effects with ComBat for the proteome, and sva-seq and limma for the transcriptome, the PCA shows that (*C*) proteomic and (*D*) transcriptomic samples group based on the presence or lack of mutation in DMD. For the (*E*) proteomics data, proteins with a log2foldchange > 0.5 and *P* < 0.5 are coloured, and for the (*F*) transcriptomics data, genes with adjusted *P* < 0.05 are coloured. Genes and proteins upregulated (UP) and downregulated (DOWN) in DMD cells are marked according to the legend below.

The principal component analyses (PCAs) of both the proteome (*Figure 3A*) and the transcriptome (*Figure 3B*) show clear pairs of samples made up of the controls and the corresponding DMD counterparts from the same donor at the same differentiation batch. This suggests that the genetic background of the donor has a strong impact on the transcriptome and proteome of these cells, as well as the differentiation batch might influence it. Once the batch and donor effects are subtracted, the PCA clearly separates the controls from the DMD cells in the proteome and transcriptome (*Figure 3C* and *D*). This means that despite the genetic background, DMD induces common changes in the transcriptomic landscape that allow the separation of controls from DMD cells based on their mRNA and protein content.

The differential expression analysis of the transcriptomic data, accounting for the batch and donor effects, revealed nine significant (adjusted *P* < 0.05) differentially expressed genes. Regarding proteomic data, no protein was found to be significantly altered (adjusted *P* < 0.05). Despite the few significant genes, we observed 761 proteins and 2641 transcripts (*Figure 3E* and *F*) with large fold changes (|log2FC| > 1 and |log2FC| > 0.5, respectively). From the list of transcripts ranked by FC, we identified 79 GO terms, and 25 KEGG pathways significantly (adjusted *P* < 0.05) altered in DMD, including JAK-STAT and Hippo pathways (see Supplementary material online, *Figure S3*).

When comparing the direction of the transcripts and proteins with a |log2FC| > 0.5, we found that 70.3% of the proteins and transcripts change in the same direction (23 up-regulated in DMD and 22 down-regulated in DMD), but 29.7% of the proteins and transcripts change in opposite directions (see Supplementary material online, *Figure S4A*). This is also reflected in our qPCR results, which correlate with the RNA-seq data (see Supplementary material online, *Figure S4B*–*F*).

The lack of few specific significant genes, but the substantial number of differentially abundant genes and proteins with large fold changes and high number of altered pathways might indicate that DMD does not always affect the same specific genes in the same manner. Rather than strong effects on a few genes, the data indicate multiple changes in the transcriptomic and proteomic landscape. Furthermore, the observed differences in the regulation of transcripts and proteins suggest that post-transcriptional regulation might play an important role in DMD.

### 3.2 DMD hiPSC-CM demonstrate a decreased level of mitoNEET protein and an increased level of both cytosolic and mitochondrial labile iron pool

Next, we closely examine those transcripts and proteins with a larger FC. Among them, mitoNEET, a protein encoded by the *CISD1* gene, with the hitherto unknown role in dystrophin-deficient cardiomyocytes, attracted our attention. This protein had a log2FC = −0.66 in the proteome [among the top 20 (*P* < 0.05; log2FC < −0.5)] (*Figure 3E*) and log2FC = 0.20 in the transcriptome (*Figure 3F*). MitoNEET, among others, regulates cellular iron homeostasis,^11^ thus we proceeded with a series of experiments described below to test the role of this protein, and iron metabolism, by extension, in dystrophin-deficient cardiomyocytes.

Taking into account the strong effect of the genetic background observed in our global analyses, to increase the robustness of our results, we prepared another isogenic pair of hiPSC (DMB03) (see Supplementary material online, *Figures S5* and *6*). However, to create this line, we decided to utilize the opposite approach, namely, to obtain the hiPSC line from a patient with a mutation in the *DMD* gene and then correct it using the CRISPR/Cas9 system (*Figure 1B*). Our intention was not only to strengthen the study by evaluation of three independent isogenic cell lines, but importantly, to test whether the restoration of dystrophin would reverse the changes caused by *DMD* mutation. Additionally, such an approach allowed us to address the issue of off-target activity of sgRNAs used to generate DMD DMB01 and DMB02 lines since to correct the mutation in the patient-specific hiPSC line (DMB03), a different sgRNA, with a distinct off-target profile, was used.

**Figure 4:**
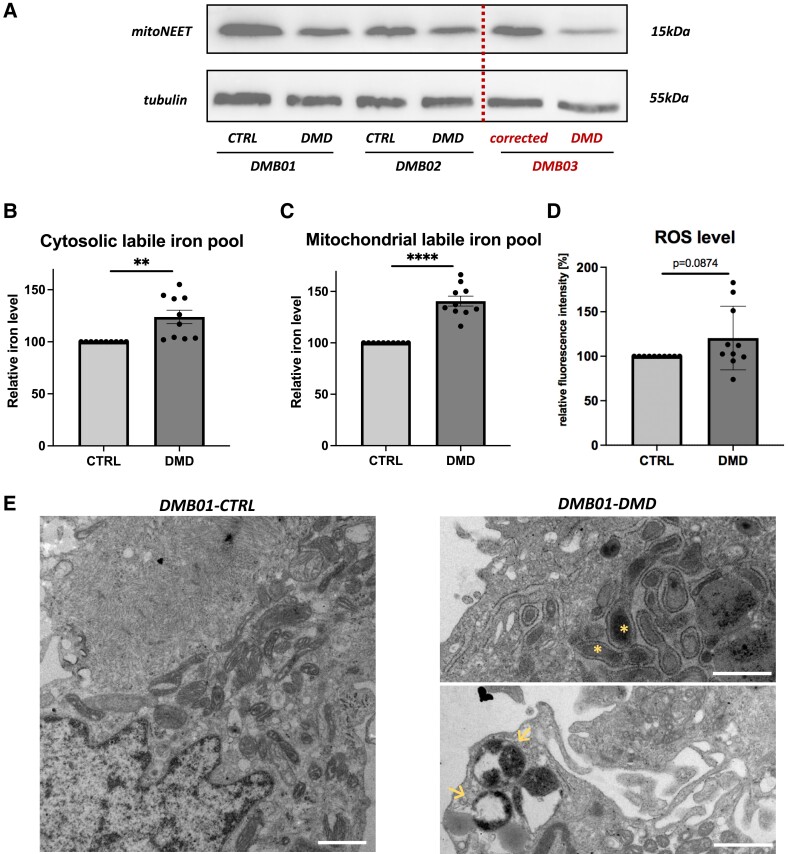
Disturbances in iron homeostasis, oxidative stress, and mitochondrial ultrastructure in DMD hiPSC-CM. (*A*) Result of western blot assessing the level of mitoNEET protein, protein that was decreased by DMD exon 50 deletion (DMB01 and DMB02) and restored by DMD gene correction in DMD patient-derived hiPSC-CM, *N* = 3, *n* = 3; (*B*) level of cytosolic labile iron pool, *N* = 3, *n* = 3; (*C*) level of mitochondrial labile iron pool, *N* = 3, *n* = 3–4; (*D*) level of ROS production, *N* = 3, *n* = 2–4; (*E*) ultrastructure images of the control and DMD DMB01 hiPSC-CM analysed by TEM demonstrating degenerated mitochondria (asterisks) and autophagosomes (arrows) in DMD hiPSC-CM. Scale bars represent 1 µm. Western blot result presented as a representative picture. Flow cytometric analysis results presented as inverted percentage of fluorescence intensity median values normalized to control = 100% (data from DMB01, DMB02, and DMB03 pooled together), **P < 0.01, ****P < 0.001, unpaired two-tailed Student’s *t*-test. LIP, labile iron pool; ROS, reactive oxygen species.

Accordingly, we confirmed the down-regulation of mitoNEET in the DMD DMB01 and DMB02 lines by western blot, and this effect was indeed reversed by gene correction in DMB03 hiPSC-CM (*Figure 4A*). Furthermore, we observed increased mitochondrial (*Figure 4B*) and cytosolic (*Figure 4C*) labile iron pool in dystrophin-deficient hiPSC-CM, as well as a tendency to increased ROS production (*Figure 4D*). Accordingly, in control cells, intact mitochondrial morphology was observed (*Figure 4E*). In contrast, DMD hiPSC-CM demonstrated areas of degenerated mitochondria with damaged mitochondrial cristae structure and autophagosomes (*Figure 4E*).

To evaluate whether the lower level of mitoNEET in DMD hiPSC-CM may be directly related to the dystrophin deficiency, we applied Enrichr software^12^ to search for transcription factors binding to *CISD1* promoter. Among them we noticed the TEAD4, which interacts with YAP transcriptional activator. The latter has been demonstrated to directly bind to dystroglycan 1, a member of dystrophin–glycoprotein complex (DGC),^13^ highlighting a possible cross-talk between dystrophin deficiency, destabilization of DGC, and altered YAP activity. Importantly, our transcriptomic analysis revealed that Hippo pathway, responsible for YAP phosphorylation and the inhibition of its nuclear translocation, is activated in DMD hiPSC-CM (see Supplementary material online, *Figure S3D*). Thus, to verify whether the expression of mitoNEET in hiPSC-CM might be regulated by Hippo-YAP signalling, we analysed YAP intracellular localization in isogenic control and dystrophin-deficient cardiomyocytes obtained from all lines used in the study. In parallel, we subjected control and dystrophin-deficient hiPSC-CM to 24 h stimulation with 10 µM TRULI, a Hippo pathway inhibitor (specifically, it inhibits LATS1 and LATS2 kinases) that favours active, unphosphorylated state of YAP and performed western blot analysis of mitoNEET level. Obtained results revealed that YAP is predominantly localized in the nucleus both in control and DMD hiPSC-CM (see Supplementary material online, *Figure S7*), thus dystrophin deficiency does not seem to influence its cellular localization. Interestingly, application of TRULI tended to increase the level of mitoNEET in DMD hiPSC-CM (*P* = 0,0538, Supplementary material online, *Figure S8A* and *B*) indicating that activation of Hippo pathway in these cells, observed in the transcriptomic analysis, may contribute to mitoNEET down-regulation. Nevertheless, the effect was not strong enough to claim that it is the main mechanism affecting the level of this protein in dystrophin-deficient cardiomyocytes. In parallel, we observed no alterations in mitoNEET after modulation of RhoA/Rho-associated protein kinase (ROCK)/Serum Response Factor (SRF) signalling pathway using ROCK inhibitor (data not shown). Thus, further research is needed to better understand the casual link between lack of dystrophin in hiPSC-CM and decreased level of mitoNEET.

### 3.3 Iron overload in DMD hiPSC-CM is associated with abnormal levels of selected factors involved in the regulation of iron metabolism

**Figure 5:**
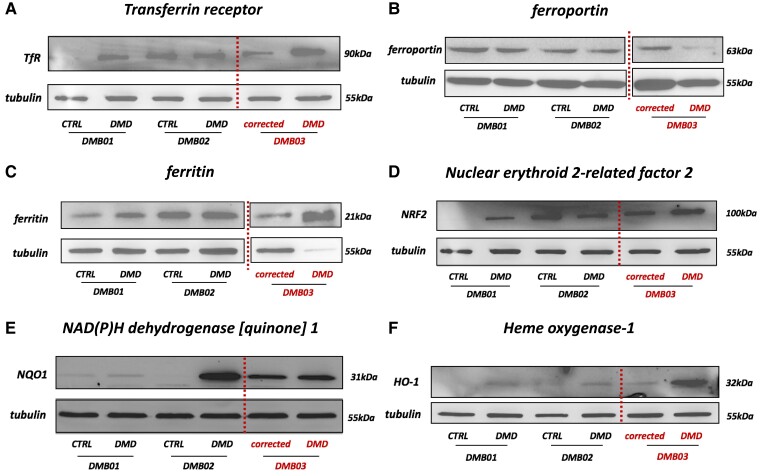
Western blot analysis of the proteins involved in the regulation of iron homeostasis. (*A*) Transferrin receptor protein level; (*B*) ferroportin protein level; (*C*) ferritin protein level; (*D*) nuclear erythroid 2-related factor 2 (NRF2); (*E*) NAD(P)H dehydrogenase [quinone] 1 (NQO1), and (*F*) heme oxygenase-1 (HO-1). Western blot results presented as representative pictures, *N* = 3, *n* = 2–3.

Iron overload in dystrophin-deficient cardiomyocytes has not been previously described, therefore, we decided to investigate the factors regulating intracellular iron homeostasis. First, we evaluated the level of proteins involved in the import and export of this microelement: transferrin receptor (TfR, encoded by the *TFRC* gene) and ferroportin (encoded by the *SLC40A1* gene), respectively. Western blot analysis demonstrated an increased level of TfR in DMB01 (*Figure 5A*). Importantly, a similar up-regulation was observed in patient-specific DMB03 hiPSC-CM, which was reversed by gene correction (*Figure 5A*). In parallel, the ferroportin protein level was decreased in DMD hiPSC-CM (*Figure 5B*). This indicates an increased import of iron into the cells with a concomitant decrease in the ability to export it, which may contribute to the observed iron overload phenotype of DMD cardiomyocytes. Interestingly, western blot analysis indicated up-regulation of the ferritin protein in these cells, both when the mutation was introduced into the control cells and when it was corrected in the patient-specific line (*Figure 5C*), which is in line with increased intracellular iron content. In addition, performed experiments revealed significantly decreased expression of *HAMP*, encoding hepcidin in DMB02 and DMB03 DMD hiPSC-CM (see Supplementary material online, *Figure S4E*), while the *TF* mRNA level, encoding transferrin was reduced in DMB02-DMD cells (see Supplementary material online, *Figure S4F*). As noted above, the qPCR analysis confirmed the similar levels of mRNA levels as observed in RNA-seq, as demonstrated also for TFRC, SLC40A1, and FTH1 mRNAs (see Supplementary material online, *Figure S4B*–*D*).

As iron accumulation was increased in DMD cardiomyocytes (*Figure 2B* and *C*) what may lead to more ROS generation (*Figure 4D*), the expression of some major antioxidant genes has been analysed. Interestingly, in DMB01-DMD, we observed up-regulation of nuclear erythroid 2-related factor 2 (NRF2), the factor regulating the level of numerous proteins involved in iron handling, as well as in the antioxidant defence (*Figure 5D*). A similar increase in NRF2 level was detected in patient-specific DMB03 cardiomyocytes and this effect was reversed by *DMD* gene correction (*Figure 5D*). Accordingly, the level of NAD(P)H dehydrogenase [quinone] 1 (NQO1) (*Figure 5E*) and heme oxygenase-1 (HO-1) (*Figure 5F*), the major antioxidant proteins regulated by NRF2, was increased in dystrophin-deficient hiPSC-CM from all three donors. These changes may indicate the protective response of DMD cardiomyocytes to oxidative stress induced by increased iron and may partially explain why the level of ROS did not change significantly in DMD cells, despite higher iron accumulation.

### 3.4 Oxidative stress in DMD hiPSC-CM can be mitigated by the use of deferoxamine and pioglitazone

**Figure 6:**
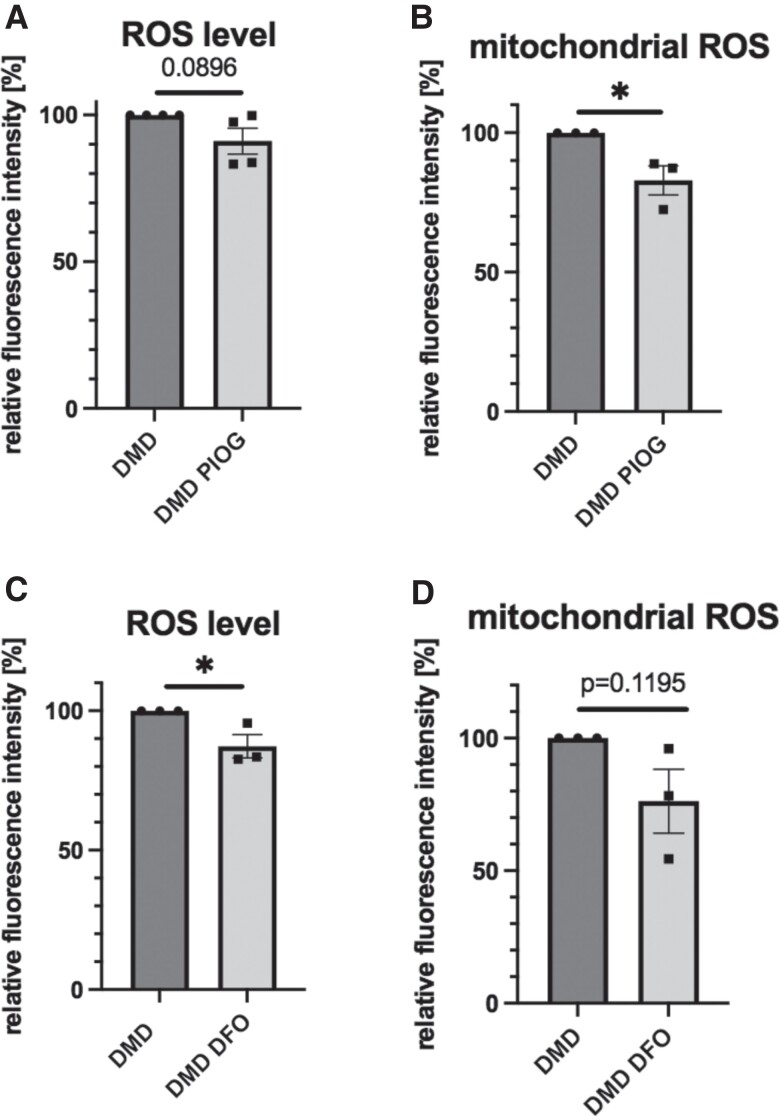
Analysis of oxidative stress in DMD hiPSC-CM by determination of (*A*) ROS and (*B*) mitochondrial ROS production level after 24 h stimulation with 10 µM pioglitazone (PIOG) and (*C*) ROS and (*D*) mitochondrial ROS production level after 2 h stimulation with 20 µM deferoxamine (DFO) using FACS. Results are presented as median (ROS) and mean (mitochondrial ROS) fluorescence intensity values normalized to DMD = 100%, *P < 0.05, *N* = 3, unpaired two-tailed Student’s *t*-test.

Subsequently, we aimed to verify whether the known iron chelator—DFO and pioglitazone, a drug targeting and stabilizing the mitoNEET protein,^14^ can affect oxidative stress in DMD hiPSC-CM. Our results demonstrated that after stimulation with pioglitazone the total ROS level tends to be decreased (*Figure 6A*), while the mitochondrial ROS level is significantly reduced (*Figure 6B*). In the case of stimulation with DFO, ROS values are significantly diminished (*Figure 6C*), while mitochondrial ROS production tends to decrease (*Figure 6D*).

### 3.5 The effect of deferoxamine and pioglitazone on electrophysiological properties of control and DMD hiPSC-CM

**Figure 7:**
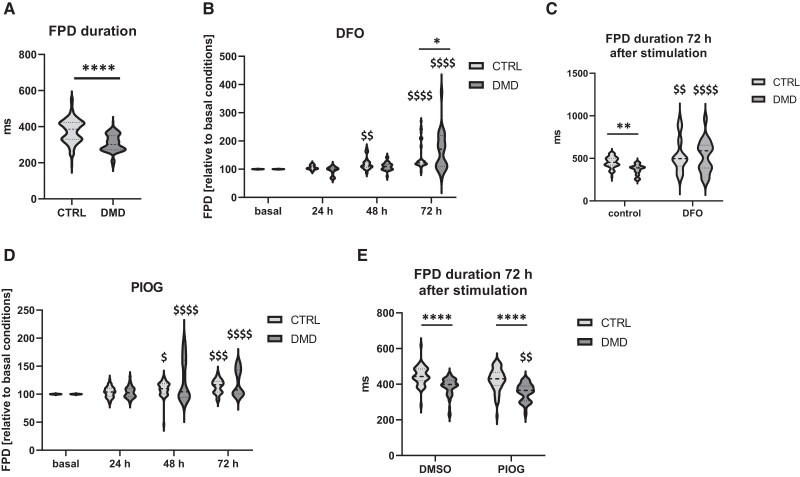
MEA analysis of the electrophysiological properties of control (CTRL) and DMD hiPSC-CM. (*A*) FPD values in basal conditions; (*B*) FPD fold change normalized to the values obtained in basal conditions in control and DMD hiPSC-CM stimulated with 20 µM deferoxamine (DFO)—24, 48, and 72 h after stimulation; ^$$^ *P* < 0.01, ^$$$$^ *P* < 0.0001, basal vs. 48 and 72 h; (*C*) FPD values in hiPSC-CM 72 h after stimulation with 20 µM DFO, unstimulated cells from the same time point served as a control; ^$$^ *P* < 0.01, ^$$$$^ *P* < 0.0001, control vs. DFO; (*D*) FPD fold change normalized to the values obtained in basal conditions in control and DMD hiPSC-CM stimulated with 10 µM pioglitazone (PIOG)—24, 48, and 72 h after stimulation; ^$^ *P* < 0.05, ^$$$^ *P* < 0.005, ^$$$$^ *P* < 0.0001, basal vs. 48 and 72 h (*E*) FPD values in hiPSC-CM 72 h after stimulation with 10 µM PIOG, unstimulated cells from the same time point served as a control; ^$$^ *P* < 0.01, DMSO vs. PIOG. In all graphs: *P < 0.05, **P < 0.01, ****P < 0.0001, CTRL vs. DMD hiPSC-CM; *N* = 2, *n* = 1–2, two-way ANOVA with Tukey’s correction for multiple comparison. Data are presented as the distribution of signals measured from all active electrodes.

In the last step, we evaluated the effect of both drugs on the electrophysiological properties of control and dystrophin-deficient hiPSC-CM using MEA analysis. Particularly, we seeded the cells from DMB01 and DMB03 lines on 24-well MEA plates, measured their basal electric activity, then stimulated with either 10 µM pioglitazone or 20 µM DFO and performed further measurements 24, 48, and 72 h after stimulation. Interestingly, in basal conditions we observed decreased values of field potential duration (FPD), corresponding to QT interval in electrocardiogram, in DMD hiPSC-CM in comparison to their control counterparts (*Figure 7A*). Other parameters, on the other hand, including the RR interval (see Supplementary material online, Figure  *S9A*), Peak-to-Peak Slope (Slope, Supplementary material online, Figure  *S9B*) and Peak-to-Peak amplitude (see Supplementary material online, Figure  *S9C*) did not differ significantly between the studied groups. More in-depth analysis revealed that with time, the FPD values, when normalized to basal recordings, increased in unstimulated (control for DFO, dissolved in the medium) (see Supplementary material online, Figure  *S9D*) and DMSO-treated cells (see Supplementary material online, Figure  *S9E*). A similar phenomenon was observed in DFO-treated hiPSC-CM (*Figure 7B*), thus, to better evaluate the long-term effect of the drug, we compared the absolute values of FPD obtained in control and DMD hiPSC-CM at this time point (*Figure 7C*). Of note, application of DFO increased the FPD in control and DMD hiPSC-CM and no significant differences were observed between both genotypes. Thus, DFO to some extent normalized the FPD in DMD hiPSC-CM, however in several recordings we noticed that it substantially prolonged the FPD in both genotypes, which should be taken into account when considering such agent for chronic application in humans. In pioglitazone-treated cells, we again observed significant changes in FPD values with time (*Figure 7D*), thus we applied a similar analysis pattern as for DFO. Interestingly, obtained results demonstrated that stimulation of DMD hiPSC-CM with this drug decreased the absolute values of FPD after 72 h (*Figure 7E*). The exact mechanism of pioglitazone-mediated effect on electrophysiological properties of DMD hiPSC-CM, however, has to be further investigated.

## 4. Discussion

The objective of this study was to investigate the mechanisms of DMD-associated cardiomyopathy, which would allow to better understand the specific causes of the heart-related complications and to identify potential therapeutic targets. For the purpose of our research, we employed a model based on hiPSC-derived cardiomyocytes. In the first step we performed the omics analyses of the control and isogenic dystrophin-deficient hiPSC-CM. Down-regulation of mitoNEET protein, an important regulator of mitochondrial iron level was observed, which together with previous finding regarding the disturbed iron metabolism and altered levels of proteins or genes closely associated with iron metabolism in dystrophin-deficient tissues^8,15,16^ prompted us to investigate more deeply the iron homeostasis in DMD hiPSC-CM. Our study for the first time demonstrated an iron overload both in the cytoplasm and the mitochondria of DMD hiPSC-CM, which provokes an increased oxidative stress and mitochondria damage. Importantly, CRISPR/Cas9 correction of *DMD* mutation in patient-derived iPSC-CMs restored the level of mitoNEET protein, and corrected other abnormalities observed in DMD hiPSC-CMs, while the application of deferoxamine (iron chelator) and pioglitazone (mitoNEET stabilizing compound) reduced ROS production in DMD hiPSC-CM.

In the first step, we compared the global transcriptomic and proteomic profiles of control and DMD cardiomyocytes and observed that the lack of dystrophin affects multiple signalling pathways in hiPSC-CM with little significant impact on specific gene expression (only nine differentially expressed genes were found with adjusted *P* < 0.05). The latter was related to the strong effect of genetic background. Our results remain in parallel with the observation of Kamdar *et al*.^17^ who noted heterogeneity in single cell RNA-seq analysis performed on DMD hiPSC-CM and a strong batch effect.

A similar outcome was observed in proteomic analysis, where multiple proteins demonstrated altered abundance; however, due to donor-based clustering of samples, none of them reached statistical significance. Accordingly, our data highlight the benefit and necessity of isogenic hiPSC line application that facilitates discrimination between the genetic background and dystrophin deficiency-associated alterations in hiPSC-CM. Interestingly, comparison of transcriptomic and proteomic data revealed that a number of proteins changed their level in a different direction than the corresponding transcripts, pointing to the important role of post-transcriptional regulation in DMD hiPSC-CM. Our qPCR and western blot analyses of the factors involved in iron homeostasis further corroborated this observation.

Importantly, despite the observed heterogeneity of the samples, changes in gene expression still translated to the common altered pathways in DMD cells, among which were those described to regulate cardiomyocyte physiology, e.g. JAK-STAT and Hippo pathways.^18,19^ Moreover, GSEA demonstrated that the products of genes with altered expression in DMD hiPSC-CM perform their functions mainly within the cell membrane, extracellular matrix, and cytoskeleton. This is certainly reasonable as dystrophin deficiency results in loss of cell membrane stability and integrity, and, as a consequence, increased susceptibility to damage. Similar observations regarding changes in connective tissue development, cell–cell junctions, and cytoskeleton organization have been demonstrated in the study comparing transcriptome of tibialis anterior muscles from murine model of DMD compared to control mice.^20^ Activation of ECM-related processes stands in line with histological evaluations of DMD muscle and cardiac samples, which demonstrated abnormal deposition of collagen and other ECM proteins manifesting the fibrosis development.^21,22^ However, our attention was attracted by the down-regulation of the mitoNEET protein in dystrophin-deficient cardiomyocytes. Of note, decreased level of this protein was detected in patient-specific DMD hiPSC-CM that was reversed by the correction of *DMD* mutation in these cells. MitoNEET, encoded by *CISD1*, was first described in 2004, when it appeared to be a target for pioglitazone, a type II diabetes drug.^6^ It is located in the outer mitochondrial membrane and plays a role in the binding of iron in the form of Fe–S clusters, as well as in the regulation of energy metabolism through their redox sensing properties.^23^ Remarkably, it has been found that among all tissues, *CISD1* has the highest expression in the heart,^24^ and its absence has been linked with the development of heart failure,^11^ nevertheless, disturbances in the regulation and function of this protein have never been described in DMD. Although we have not deciphered the exact mechanism responsible for the decrease in mitoNEET level in dystrophin-deficient cardiomyocytes, we observed its modest up-regulation after inhibition of the Hippo pathway, found to be activated in DMD hiPSC-CM in transcriptomic analysis. Of note, activation of this pathway was previously observed in skeletal muscle specimens derived from DMD patients,^25^ however the downstream signalling that regulates mitoNEET abundance has to be further investigated. Interestingly, Ham *et al.*^26^ demonstrated recently that mitoNEET is a substrate for Parkin-mediated ubiquitination and subsequent degradation. Thus, our previous observation that the level of Parkin is increased in diaphragm of dystrophin-deficient mice (*mdx* strain)^27^ may highlight another possible pathway involved in mitoNEET regulation also in DMD hiPSC-CM. Accordingly, in a parallel study (Martyniak *et al.* in preparation), we detected in these cells disturbances in the mitophagy process in which the Parkin is involved, however, the direct link between dystrophin deficiency and the alterations in mitochondrial quality control has to be still deciphered in human cardiomyocytes.

One of the effects of decreased mitoNEET protein level is excessive mitochondrial iron accumulation.^11^ Importantly, our analyses revealed a significant increase in free iron concentration in both mitochondria and the cytoplasm of DMD cardiomyocytes. Commonly, iron overload is associated with several negative effects, such as ROS production, cellular damage, and cell death.^28^ The mechanism underlying these effects is predominantly related to an excessive free, unbounded iron pool, which enters the Fenton reaction, the product of which are hydroxyl radicals.^29^ Accordingly, we noted the altered intracellular structure and elevated ROS production by DMD cardiomyocytes.

Interestingly, we found also a higher level of TfR protein in DMD hiPSC-CM, what might be linked to increased iron uptake.^30^ Moreover, a decreased level of ferroportin, the main iron exporter,^31^ was observed in DMD cardiomyocytes. Correspondingly, mutations in the *SLC40A1*, the gene encoding ferroportin, have previously been reported and associated with iron accumulation.^32,33^ The main storage protein, ferritin, was also up-regulated in dystrophin-deficient hiPSC-CM, being a probable defence mechanism to bind free iron ions and decrease the harmful abundance of labile iron pool in the cytoplasm. Accordingly, increased ferritin levels were previously observed in iron overload diseases, where the storage proteins, including both ferritin and transferrin, are abundant/saturated and serve as a diagnostic sign.

Besides transferrin-bound iron entry, another source of iron in the cytoplasm is heme. In the next step, we thus evaluated the levels of NRF2, a protein involved in heme metabolism, and HO-1, an antioxidant enzyme, which catalyses the degradation of heme to biliverdin, carbon monoxide, and ferrous iron.^34^ HO-1 is a direct target of NRF2, and both proteins are inextricably linked to iron homeostasis.^34^ Interestingly, both NRF2 and HO-1 were found to be up-regulated in DMD hiPSC-CM, including patient-specific cardiomyocytes in which gene correction reversed the level of both proteins. Such up-regulation might be in turn related to only non-significant increase in ROS production in DMD cardiomyocytes in our experiment, due to the antioxidant effect of HO-1,^34^ as well as increased level of NQO1, which is known to play a protective role against oxidative stress.^35^ Importantly, excessive oxidative stress has been previously demonstrated in various *in vitro* and *in vivo* models of DMD^7,36^ and the underlying triggers identified so far included severe calcium influx, mitochondria dysfunction, and NOX enzymes activation.^7,36,37^ Here, we demonstrate for the first time a novel mechanism contributing to a disbalanced redox state in dystrophin-deficient human cardiomyocytes. In fact, the relation between oxidative stress and iron overload in DMD was considered already in 1984, when Clark^38^ proposed deferoxamine, a potent iron chelator, as a treatment for DMD patients, however, since then, this issue has not been a subject of thorough further investigation.

A link between iron overload and the cardiomyopathy development has been frequently reported in the literature.^39,40^ Usually, it results from systemic disturbances in iron balance, such as excessive intestinal absorption or/and aberrant iron storage. Similarly, as in the case of DMD-associated cardiomyopathy, cardiac iron overload is virtually not manifested in early age and the first symptoms usually appear in middle-aged patients. This is due to the fact that the accumulation develops gradually and first occurs in the epicardium, while later is observed also in the myocardium and endocardium.^39^ Our study, in turn, indicates an intrinsic disturbance of iron metabolism in human DMD cardiomyocytes. It is of high relevance, as cardiac iron overload significantly affects the proper functioning of the heart^39^ and one of the most specific signs of iron overload is the shortening of the T2 relaxation time that is inversely correlated with the level of iron accumulation.^41,42^ Interestingly, the available cardiac magnetic resonance imaging results performed in DMD patients indicate on decreased T2 relaxation time,^43,44^ although this observation has never been linked to iron metabolism disorders.

Beside the endorsed direct consequences of excessive labile iron pool on heart failure, there is another potential mechanism of impaired electrical conduction in iron-overloaded hearts. Namely, when saturated transferrin does not maintain the excessive iron transportation, the non-transferrin-bound iron can enter the cell through L-type calcium channels (LTCC), what may disturb the normal calcium transportation and, as a result, provoke the excitation-contraction coupling impairment.^40,45^ This explanation stands in line with defective calcium oscillations in DMD hiPSC-CM demonstrated in previous studies.

Notwithstanding, there are very few research papers in which iron homeostasis disturbance has been described in DMD. Bornman *et al*.^46^ demonstrated that dietary iron restriction resulted in a decreased level of Hsp70, a reduced number of macrophage-invaded necrotic fibres and a decreased creatine kinase level in *mdx* mice. Also, previous proteomic analysis of liver, heart, and skeletal muscle samples collected from *mdx* mice revealed increased ferritin levels,^16,47,48^ although no further research has been undertaken in this direction. This topic was raised again recently in the study on iron overload in the muscles of *mdx* and double dystrophin and utrophin (*mdx/utrn^−/−^*) knockout mice and its detrimental effect on muscle function.^8^ Of note, the authors applicated iron chelation treatment using deferiprone, which ameliorated the muscle pathology, decreased ROS production, and improved mitochondrial function.^8^

In our study, we verified the effect of another iron chelator—DFO, as well as pioglitazone, the drug stabilizing the mitoNEET protein, and commonly used to treat diabetes.^14^ Our results demonstrated that treatment of DMD hiPSC-CM with DFO and pioglitazone decreased the ROS level and mitochondrial ROS production, respectively. It, thus, suggests that these drugs could be considered for further evaluation as potential therapeutic agents, however more in-depth analysis of their effect on electrophysiological activity of the human cardiomyocytes should be taken into account. Yet, DMD patients develop diabetic syndromes including insulin resistance that further potentiates the possible clinical benefit of pioglitazone application as DMD treatment.^49^ Accordingly, the abnormalities in iron metabolism were reversed after correction of the mutation in DMD. The therapeutic strategy based on the restoration of dystrophin expression is also often considered as potential treatment method for patients with DMD. It is hampered by the need to reach all affected tissues, which is challenging in the case of skeletal, respiratory, and heart muscles. However, significant progress has been made recently in this matter, as FDA approved conditionally the gene therapy relying on delivery of the AAV encoding microdystrophin.^50^

Pioglitazone is an agonist of peroxisome proliferator-activated receptor gamma (PPARγ) and a known insulin sensitizer, primarily used to treat type 2 diabetes. Besides diabetes, pioglitazone has been also tested for treatment of other conditions, such as Alzheimer’s disease, cancers, proteinuria, non-alcoholic fatty liver disease and Parkinson’s disease showing positive effects in both animal and pre-clinical studies, suggesting its multiple targets and pleiotropic benefits, mostly related to its anti-inflammatory properties.^51,52^ However, further detailed studies are required to validate pioglitazone safety, as in the past strong cardiovascular side effects have been identified after treatment with rosiglitazone, another representative of thiazolidinediones. They included increased risk of myocardial infarction, stroke, heart failure, and even death^53^ and resulted in its withdrawal in 2010 from European and US market. In case of pioglitazone itself, both positive^54,55^ and negative effects on heart function have been observed,^56^ however due to concerns related to increased bladder cancer incidence, FDA added restrictions on the prescribing of pioglitazone, while in some countries the drug has been completely discontinued. Despite many studies to date, the effect of thiazolidinediones on heart function is inconclusive, therefore safety concerns related to their potential therapeutic use should be first elucidated.

In our previous study, performed on mouse *mdx* model of DMD, we have found disturbances in mitochondrial metabolism and mitophagy.^27^ Here we show the presence of autophagosomes, cytoplasmic vacuolation, and degenerating mitochondria with dissolution of mitochondrial cristae in DMD cardiomyocytes indicating on ongoing autophagic processes and mitochondria malfunctioning. Similar observations were reported in the aforementioned study by Furihata *et al*.^11^ who described a reduced cristae density and disturbed integrity of the mitochondrial outer membrane in cardiac-specific mitoNEET KO mice, a left ventricular dysfunction, extensive interstitial fibrosis, development of heart failure, and premature death.

### 4.1 Conclusion

Our work has led us to the conclusion that DMD hiPSC-CM demonstrated impaired iron homeostasis resulting in cytosolic and mitochondrial iron overload and increased oxidative stress. Moreover, the application of DFO and pioglitazone resulted in reduced ROS production, what could be of help for future investigations of new therapeutic approaches for the amelioration of DMD cardiomyopathy. The data described here strongly indicate the direct relationship between the occurrence of iron overload and the presence of dystrophin, as the results obtained on the lines with the introduced mutation in *DMD* (DMB01 and DMB02) are consistent with the results acquired on the isogenic line created by correction of the mutation diagnosed in DMD patient (DMB03), where we observed a reversal of the effect and restoration of normal iron homeostasis.

### 4.2 Study limitations

Finally, a number of potential limitations should be considered. First, in our study, we used hiPSC-CM, which after the standard differentiation protocol have an immature phenotype. This means that some pathological features associated with the disease of interest may not manifest in the patients’ cardiomyocytes. On the other hand, the fact that the changes are already visible at this stage in dystrophic cardiomyocytes is important because the iron level in the cell will increase with age and, therefore, early clinical intervention can be beneficial. Moreover, the 2D cellular model is devoid of some physiological and microenvironmental conditions. Although our study cannot univocally confirm that iron overload is the main cause of DMD-associated pathogenesis, we believe that further research on this topic may provide novel therapeutic targets for better management of cardiac deterioration in DMD patients. Therefore, more experimental investigations are needed to validate the evidence from our studies. In particular, it would be beneficial to perform analogical experiments on animal models of DMD to investigate the iron metabolism abnormalities in dystrophic heart and to test the effectiveness of DFO and pioglitazone in vivo.

## Supplementary material

Supplementary material is available at *Cardiovascular Research* online.

## Authors’ contributions

K.A. and J.S. performed experiments. D.P. performed the proteome analysis. O.W. prepared the samples for TEM imaging. G.M. and G.Y. analysed the transcriptome and proteome data. A.M. assisted with hiPSC-CM differentiation and data collection. M.K. and E.P. helped with data interpretation. A.P.-C. and A.K.-P. recruited the patients. K.A., J.S., and J.D. designed the experiments. K.A., J.S., and J.D. prepared figures and wrote the manuscript. A.Ł. and J.D. analysed the data and reviewed the manuscript. J.S. and J.D. supervised the work. All authors reviewed and accepted the manuscript.

## Supplementary Material

cvad182_Supplementary_Data

## Notes

Translational perspective DMD-related cardiomyopathy is associated with the occurrence of iron overload and the resulting oxidative stress and mitochondrial damage. Abnormalities in iron homeostasis maintenance and the resulting iron overload may explain previously observed pathological features of DMD-associated cardiomyopathy, such as increased oxidative stress, and serve as a starting point for the search for new therapeutic strategies. Deferoxamine and pioglitazone, FDA-approved drugs for the treatment of iron overload and diabetes, respectively, demonstrated a positive effect on the reduction of ROS level in dystrophin-deficient cardiomyocytes; therefore, we believe that it would be beneficial to continue the studies to validate their effectiveness and safety in the context of DMD.
