# Supplementary material for: Dysregulated iron homeostasis in dystrophin-deficient cardiomyocytes: correction by gene editing and pharmacological treatment
Source: Cardiovasc Res. 2023 Dec 12;120(1):69–81. doi: 10.1093/cvr/cvad182 (PMC10898935; doi:10.1093/cvr/cvad182)
Supplement: cvad182_Supplementary_Data [file cvad182_supplementary_data.zip › Supplementary Material_revised.docx]

**Supplementary Material**

**Dysregulated iron homeostasis in dystrophin-deficient cardiomyocytes: correction by gene editing and pharmacological treatment**

Kalina Andrysiak *PhD*^1^, Gabriela Machaj *PhD*^2^, Dominik Priesmann *MSc*^3^, Olga Woźnicka *PhD*^4^, Alicja Martyniak *PhD*^1^, Guillem Ylla *PhD*^2^, Marcus Krüger *PhD*^3^, Elżbieta Pyza *PhD*^4^, Anna Potulska-Chromik *MD, PhD*^5^, Anna Kostera-Pruszczyk *MD, PhD^5^*, Agnieszka Łoboda, *PhD*^1^, Jacek Stępniewski *PhD*^1*^, Józef Dulak *PhD^1^*^*^

^1^Department of Medical Biotechnology; Faculty of Biochemistry, Biophysics and Biotechnology, Jagiellonian University, Kraków, Poland

^2^Laboratory of Bioinformatics and Genome Biology, Faculty of Biochemistry, Biophysics and Biotechnology, Jagiellonian University, Kraków, Poland

^3^Institute for Genetics, Cologne Excellence Cluster on Cellular Stress Responses in Aging-Associated Diseases, University of Cologne, Cologne, Germany

^4^Department of Cell Biology and Imaging, Institute of Zoology and Biomedical Research, Faculty of Biology, Jagiellonian University, Kraków, Poland

^5^ Department of Neurology, Medical University of Warsaw, Warsaw, Poland

* co-corresponding authors

**Supplementary Figures**

***Supplementary Fig. 1.*** *CRISPR/Cas9-based generation of isogenic hiPSC cell lines.* ***a.*** *PCR genotyping of edited DMB02 hiPSC after nucleofection and puromycin selection.* ***b.*** *PCR genotyping of individual DMB02 hiPSC clones. Clone marked in dashed rectangles were selected for further studies.*

*
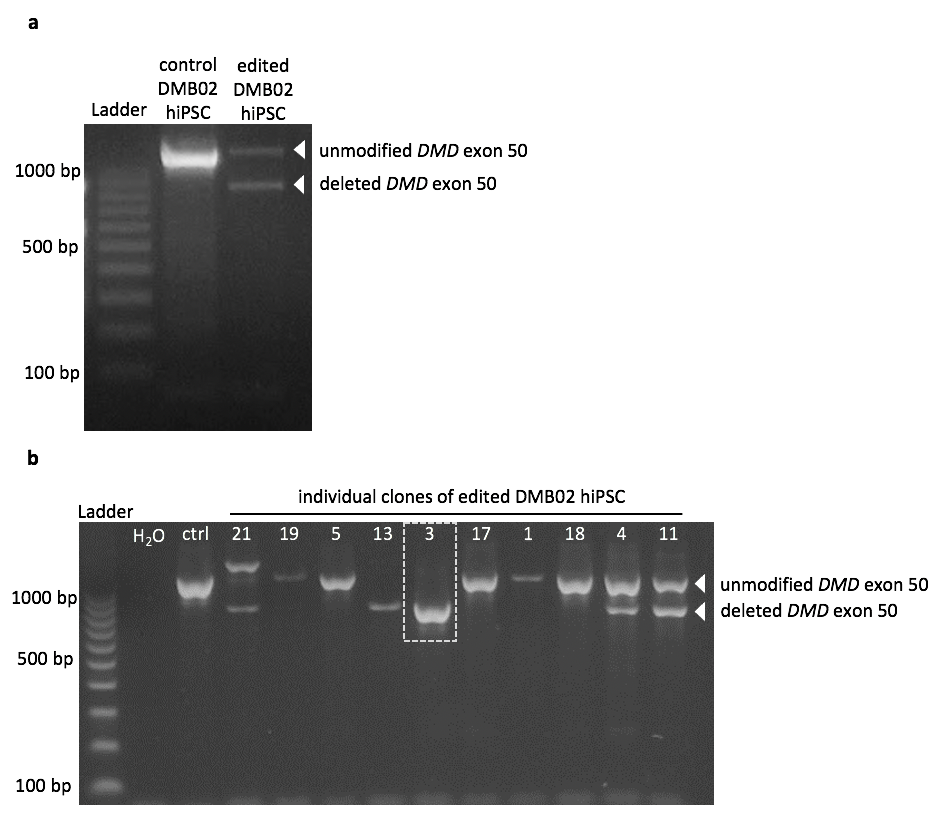
*

***Supplementary Fig. 2.*** *Characterization of DMB02 hiPSC****. a.*** *Pictures of DMB02 hiPSC lines stained for markers of pluripotency: OCT4, SSEA-4, TRA-1-60, TRA-1-81 and NANOG. Scale bars represent 100 µm;* ***b.*** *Pictures of DMB02 hiPSC lines stained for alkaline phosphatase activity. Scale bars represent 50 µm.* ***c.*** *Pictures of embryoid bodies spontaneously differentiated from the DMB02 hiPSC line used in the study, stained for markers of cells originating from all three germ layers: GATA4 (mesoendoderm), NFH (ectoderm) and αSMA (mesoderm) and vimentin (mesoderm/endoderm); nuclei were stained in blue. Scale bars represent 100 µm;* ***d.*** *Results of the karyotype analysis of DMB02 hiPSC lines.*

*
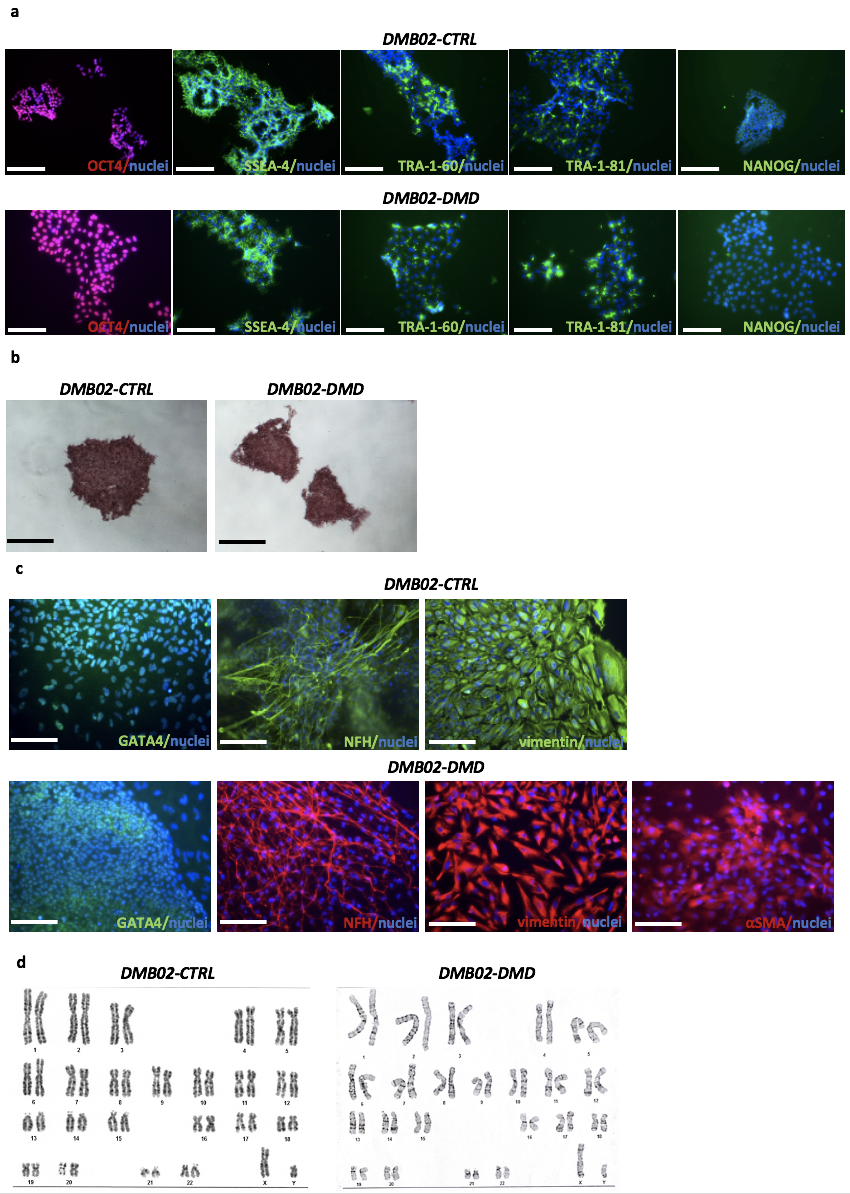
*

***Supplementary Fig. 3.*** *Dot plots of GSEA based on their assignment to* ***a.*** *Biological processes (BP) in which they are engaged (53 significantly enriched BP in total);* ***b.*** *Cellular components in which gene products perform their function;* ***c.*** *Molecular functions of the gene products;* ***d.*** *Enriched pathways (Kyoto Encyclopedia of Genes and Genomes - KEGG pathways) in which regulation they participate in (25 significantly enriched pathways in total); color of the dots corresponds to the significance level, while the size of the dot corresponds to the number of genes involved in the individual component.*

*
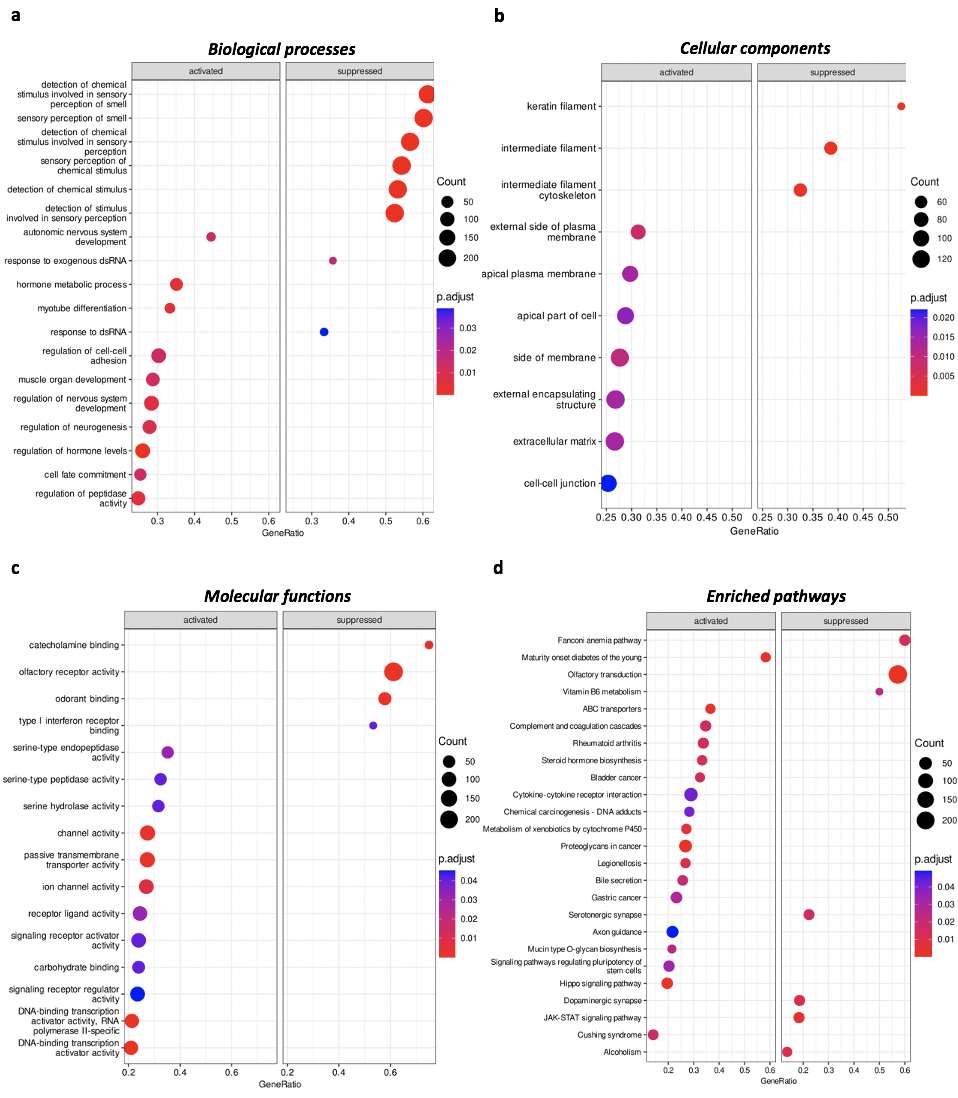
*

***Supplementary Fig. 4.*** *Coverage of obtained transcriptomic and proteomic data.* ***a.*** *UpSet plot showing the number of genes whose transcripts and protein levels display an absolute log2foldchange >0.5 in DMD compared to controls. The vertical bars indicate the number of genes that are increased in the proteome and transcriptome, the number of genes that are downregulated in both, and the number of genes that are upregulated one and low in the other. Comparison of the changes in the expression of* ***b.*** *TFRC,* ***c.*** *SLC40A1,* ***d.*** *FTH1,* ***e.*** *HAMP, and* ***f.*** *TF mRNA obtained from RNA-seq data (left panel) and qPCR analyses (right panel). The qRT-PCR results presented as relative expression normalized to control = 1, N=3, n=2-3, *p < 0.05, **p < 0.01, ***p < 0.005, unpaired two-tailed Student’s t-test.*

*
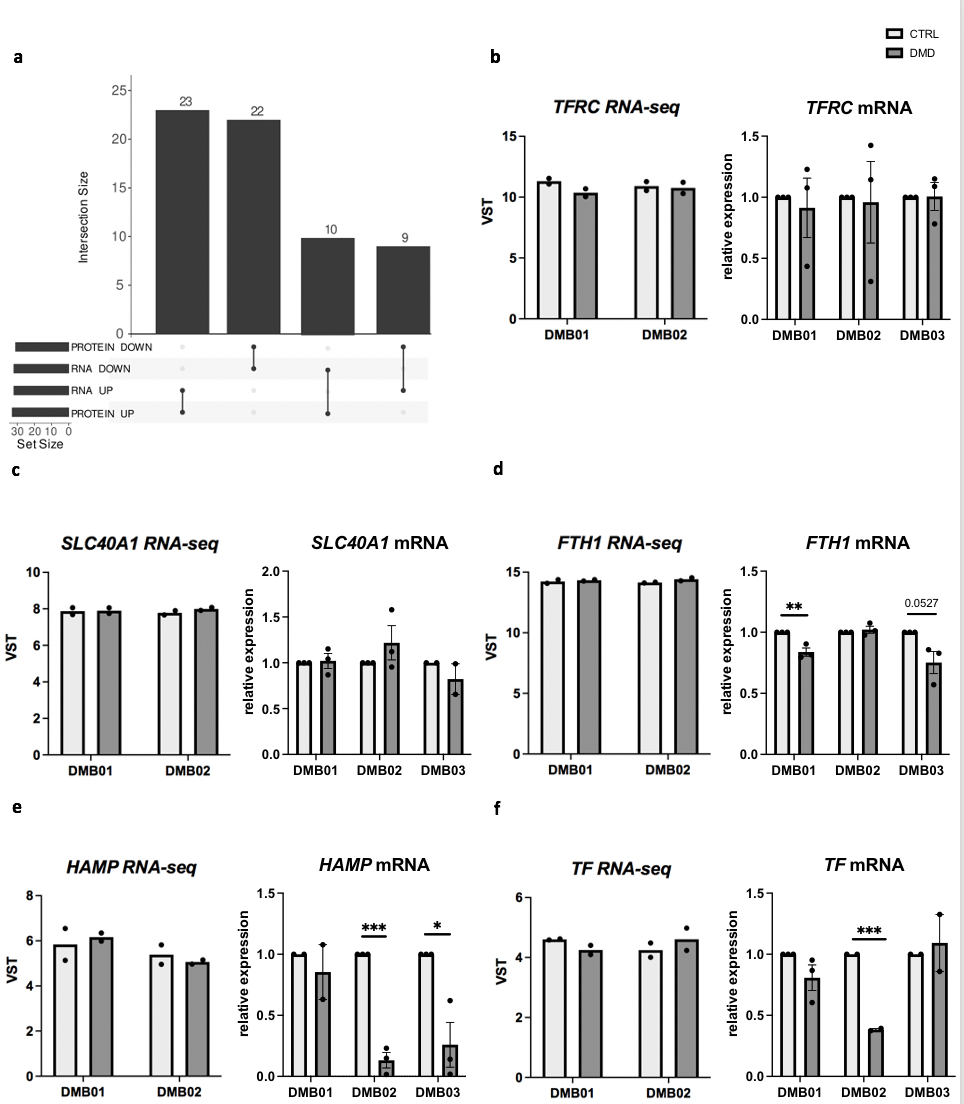
*

***Supplementary Fig. 5.*** *CRISPR/Cas9-based generation of isogenic hiPSC cell lines.* ***a.*** *PCR genotyping of DMB03 hiPSC after nucleofection and puromycin selection.* ***b.*** *PCR genotyping of individual DMB03 hiPSC clones. The clone marked in dashed rectangle was selected for further studies.*

*
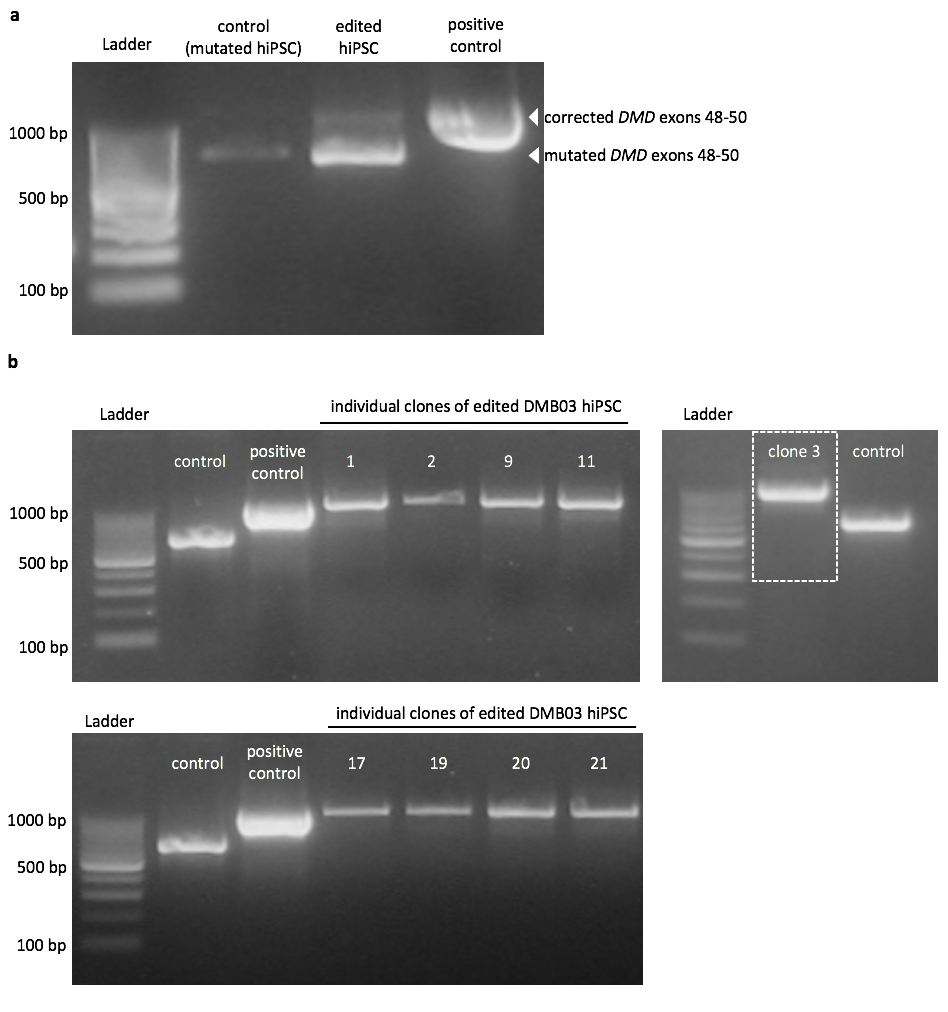
*

***Supplementary Fig. 6.*** *Characterization of DMB03 hiPSC****. a.*** *Pictures of DMB03 hiPSC lines stained for markers of pluripotency: OCT4, SSEA-4, TRA-1-60, TRA-1-81 and NANOG. Scale bars represent 100 µm;* ***b.*** *Pictures of DMB03 hiPSC lines stained for alkaline phosphatase activity. Scale bars represent 50 µm.* ***c.*** *pictures of embryoid bodies spontaneously differentiated from DMB03 hiPSC line used in the study, stained for markers of cells originating from all three germ layers: GATA4 (mesoendoderm), NFH (ectoderm) and αSMA (mesoderm) and vimentin (mesoderm/endoderm); nuclei were stained in blue. Scale bars represent 100 µm;* ***d.*** *Results of the karyotype analysis of the DMB03 hiPSC lines;* ***e.*** *Western blot analysis of dystrophin in control and DMD DMB03 hiPSC-CM shown as representative pictures (n=2, separate differentiation runs).*

*
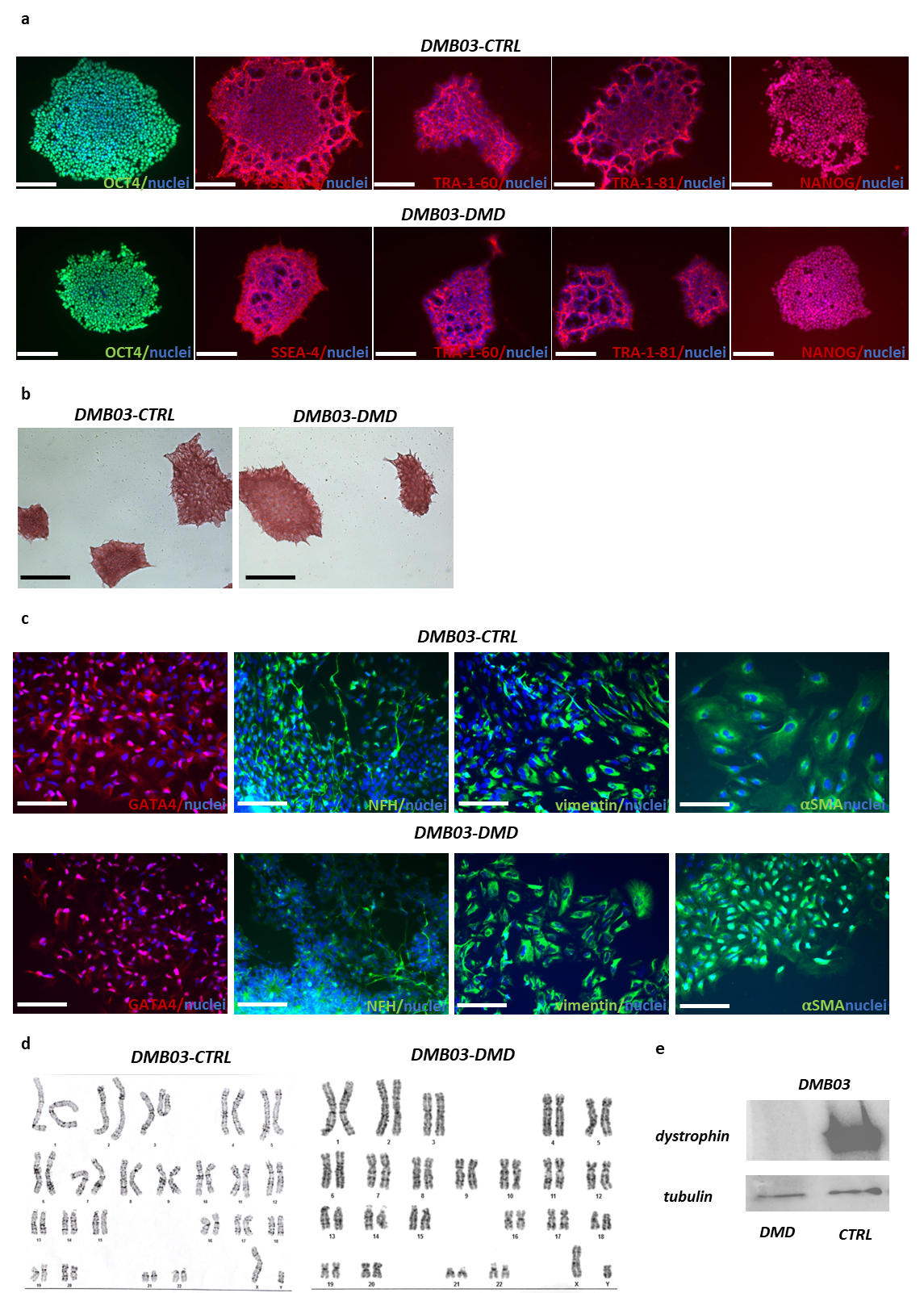
*

***Supplementary Fig. 7.*** *Immunofluorescent analysis of YAP (green) and cTnT (red) in control (CTRL) and DMD hiPSC-CM derived from DMB01 (upper panel), DMB02 (middle panel) and DMB03 (bottom panel) lines. Nuclei were stained in blue. Scale bars represent 100 µm.*


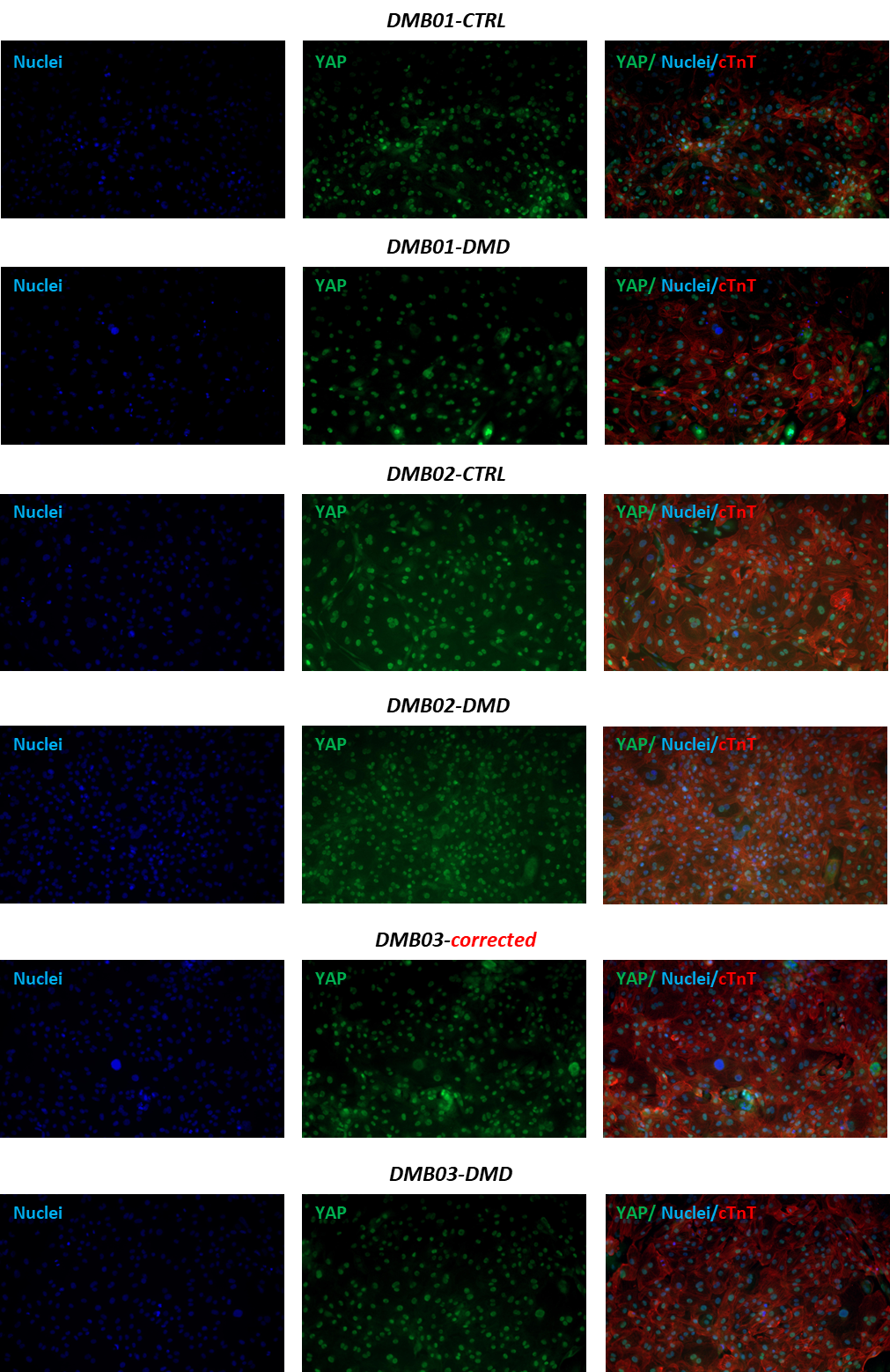


***Supplementary Fig. 8.*** *Western blot analysis of mitoNEET in DMD hiPSC-CM.* ***a.*** *Representative result from DMB01-DMD line) after 24 h stimulation with 10 µM TRULI.* ***b.*** *Results obtained from DMD hiPSC-CM derived from all three lines used in the study: DMB01, DMB01 and DMB03 were subjected to densitometric quantification. Cells treated with DMSO, a solvent for TRULI, served as a control. Tubulin was used as a protein loading control.*  *N=3, n=1-2, p=0.0538, unpaired two-tailed Student’s t-test.*

*
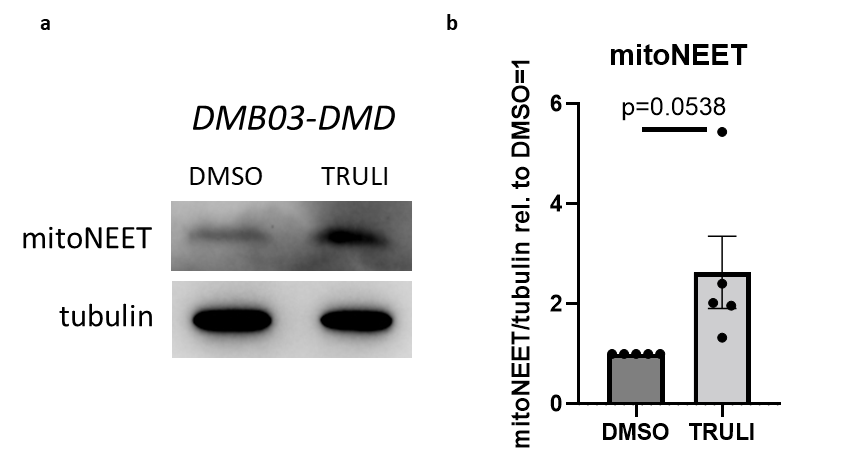
*

***Supplementary Fig. 9.*** *MEA analysis of the electrophysiological properties of control (CTRL) and DMD hiPSC-CM.* ***a.*** *RR interval values in basal conditions;* ***b.*** *Peak-to-Peak Slope (Slope) values under basal conditions;* ***c.*** *Peak-to-peak amplitude values under basal conditions;* ***d.*** *Field protein duration (FPD) fold change normalized to the values obtained under basal conditions in unstimulated control and DMD – 24 h, 48 h and 72 h after stimulation;* ***e.*** *FPD fold change normalized to the values obtained under basal conditions in control and DMD hiPSC-CM treated with DMSO – 24 h, 48 h and 72 h after stimulation. ****p < 0.0001, CTRL vs DMD hiPSC-CM; ^$$$^p < 0.005, ^$$$$^p < 0.0001, basal vs 48 h and 72 h. N=2, n=1-2, two-way ANOVA with Tukey’s correction for multiple comparison.* *Data are presented as the distribution of signals measured from all active electrodes.*

*
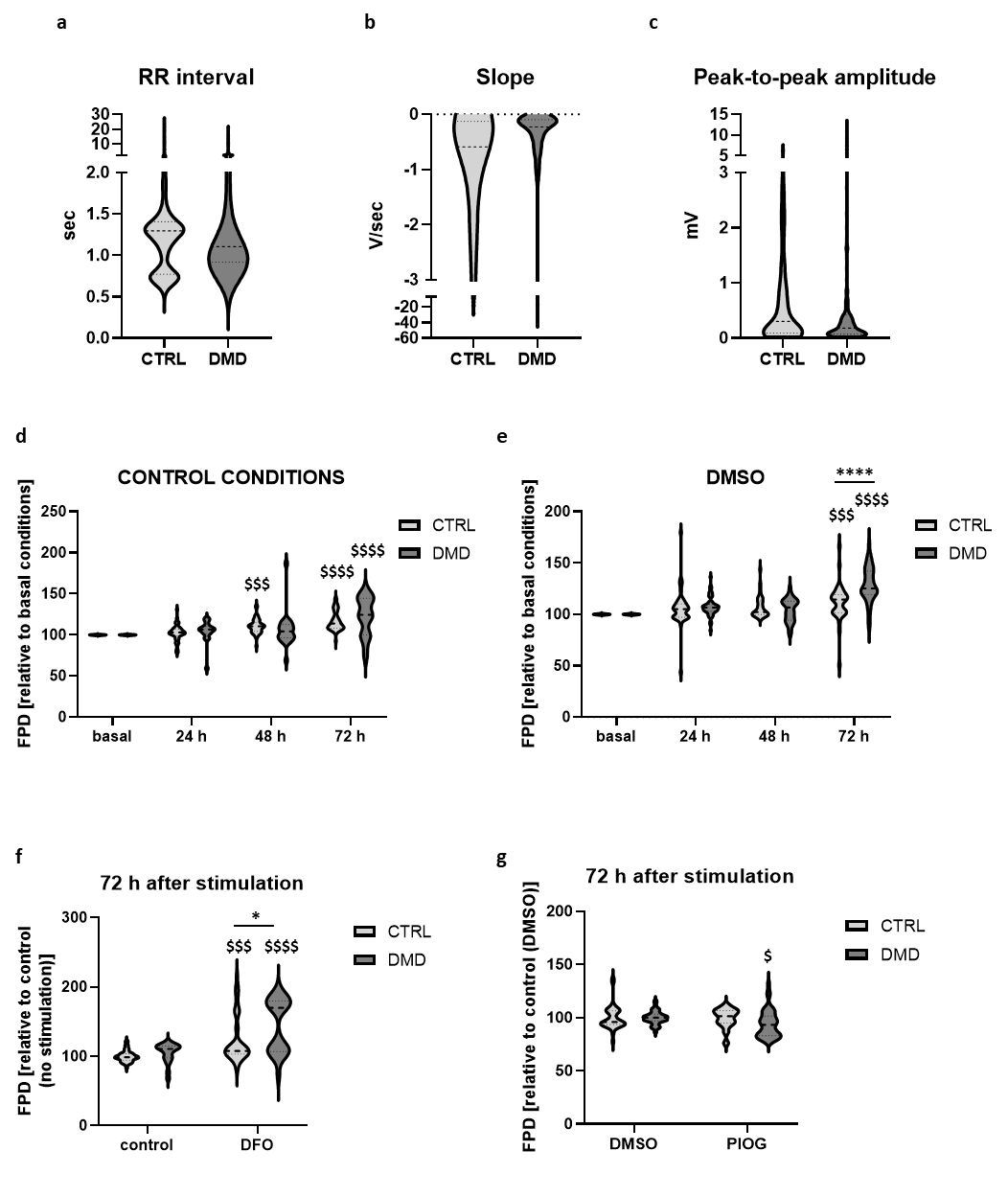
*

**Supplementary Tables**

***Supplementary Table 1.*** *Characteristics of the PBMC donors from which the hiPSC lines used in the study were obtained. The DMB01-CTRL hiPSC line was obtained and described by our group earlier*^1^.

| **hiPSC line name** | **age of the donor** | **sex** | **healthy/DMD** |
| --- | --- | --- | --- |
| DMB01-CTRL^1^ | 55 | M | healthy |
| DMB02-CTRL | 48 | M | healthy |
| DMB03-DMD | 9 | M | DMD (deletion of exons 48-50 of *DMD*) |

***Supplementary Table 2.*** *Template sequences encoding the specificity-providing fragments of tested sgRNAs.*

|  | **sgRNAs** |
| --- | --- |
| sgRNA 1 | 5’-AATGTCTAACCTTTATCCAC-3’ |
| sgRNA 2 | 5’-AACACATGTGACGGAAGAGA-3’ |
| sgRNA 3 | 5’-GACAAATCTCCAGTGGATAA-3’ |

***Supplementary Table 3.*** *Sequences of primers used in the Surveyor nuclease assay.*

|  | ***PRIMERS*** | |
| --- | --- | --- |
| ***TARGET*** | ***FORWARD*** | ***REVERSE*** |
| *DMD exon 50 5’ site* | 5’-CCCTTCAAACTTGTCCTGCTTTGAG-3’ | 5’-CACCCAGTCATCACTTCATAGTTGC-3’ |
| *DMD exon 50 3’ site* | 5’-AAGCTAGTTGCTGAGAGGGAACTGT-3’ | 5’- TTGTCCTTCCACTCTCAGGCAGC-3’ |
| *DMD exon 47-50* | 5’-AAGGTAGTTGGAATTGTGCTG-3’ | 5’-CTTGTGGCGAACGTTAGTGTAC-3’ |

***Supplementary Table 4.*** *Primary and secondary antibodies used for immunofluorescent staining.*

| **ANTIBODY** | **COMPANY** | **DILUTION** |
| --- | --- | --- |
| *PRIMARY ANTIBODIES* | | |
| OCT3/4 (goat) | *Santa Cruz Biotechnology* | 1:200 |
| TRA-1-60 (mouse) | *Sigma-Aldrich* | 1:200 |
| TRA-1-81 (mouse) | *Sigma-Aldrich* | 1:200 |
| NANOG (mouse) | *Santa Cruz Biotechnology* | 1:200 |
| SSEA-4 (mouse) | *Santa Cruz Biotechnology* | 1:200 |
| vimentin (rabbit) | *Abcam* | 1:200 |
| αSMA (rabbit) | *Abcam* | 1:200 |
| GATA4 (mouse) | *Santa Cruz Biotechnology* | 1:200 |
| NFH (rabbit) | *Abcam* | 1:400 |
| troponin T (mouse) | *Thermo Fisher Scientific* | 1:200 |
| dystrophin (rabbit) | *Abcam* | 1:100 |
| YAP (Rabbit) | *Cell Signaling Technology* | 1:200 |
| *SECONDARY ANTIBODIES* | | |
| AF488 Goat Anti-Mouse IgG | *Thermo Fisher Scientific* | 1:400 |
| AF488 Rabbit Anti-Goat IgG | *Thermo Fisher Scientific* | 1:400 |
| AF568 Goat Anti-Rabbit IgG | *Thermo Fisher Scientific* | 1:400 |
| AF488 Donkey Anti-Rabbit IgG | *Thermo Fisher Scientific* | 1:400 |
| AF568 Donkey Anti-Goat IgG | *Thermo Fisher Scientific* | 1:400 |
| AF568 Donkey Anti-Mouse IgG | *Thermo Fisher Scientific* | 1:400 |

***Supplementary Table 5****. Scheme of the qRT-PCR reaction.*

| **STEP** | **initial denaturation** | **denaturation** | **annealing** | **extension** | **melt curve** | | |
| --- | --- | --- | --- | --- | --- | --- | --- |
| **TIME** | 10 min | 30 s | 1 min | 45 s | 15 s | 1 min | 15 s |
| **TEMPERATURE [°C]** | 95 | 95 | 60 | 72 | 95 | 60 | 95 |
| **NUMBER OF CYCLES** | 1 | 40 | | | 1 | 1 | 1 |

***Supplementary Table 6.*** *List of primers used in the study and their sequences.*

| **Gene name** | **Sequences** | |
| --- | --- | --- |
|  | **Forward** | **Reverse** |
| *TNNT2* | 5’-ATCCAGAACGCCCAGACAGA-3’ | 5’-GCTGCTTGAACTTCTCCTGC-3’ |
| *FTH1 (primers’ sequences taken from Chang et al.*^2^*)* | 5’-GCCAGAACTACCACCAGGAC-3’ | 5’-TGAAGGAAGATTCGGCCACCT-3’ |
| *SLC40A1 (primers’sequence taken from Wu et al.*^3^*)* | 5’-AACAAGCACCTCAGCGAGAG-3’ | 5’-CACATCCGATCTCCCCAAGT-3’ |
| *HAMP (primers’ sequence taken from Sikorska et al.*^4^*)* | 5’-GACCAGTGGCTCTGTTTTCC-3’ | 5’-CAGGGCAGGTAGGTTCTACG-3’ |
| *TF*^2^ | 5’-AACGTAACTGACTGCTCGGG-3’ | 5’-CTGCGTTCCCATCTTCACCT-3’ |
| *TFRC*^2^ | 5’-TCGGAGAAACTGGACAGCAC-3’ | 5’-ATCACGCCAGACTTTGCTGA-3’ |

***Supplementary Table 7.*** *List of antibodies used in Western blot.*

| **Antibody** | **Company** | **Dilution** |
| --- | --- | --- |
| PRIMARY ANTIBODIES | | |
| α-Tubulin Monoclonal antibody (mouse) | *Sigma-Aldrich* | 1:1000 |
| NCL-DYSB Novocastra™ Lyophilized Mouse Monoclonal Antibody (anti-dystrophin) | *Leica Biosystems* | 1:50 |
| Ferroportin Polyclonal Antibody (rabbit) | *Thermo Fisher Scientific* | 1:500 |
| CISD1 Monoclonal Antibody (OTI2B3) (mouse) | *Thermo Fisher Scientific* | 1:500 |
| HO-1 Polyclonal antibody (rabbit) | *Enzo Life Sciences* | 1:1000 |
| Ferritin (rabbit) | *Thermo Fisher Scientific* | 1:500 |
| NAD(P)H dehydrogenase [quinone] 1 (rabbit) | *Abcam* | 1:1000 |
| NRF2 antibody (rabbit) | *Abcam* | 1:500 |
| Transferrin Receptor (mouse) | *Thermo Fisher Scientific* | 1:1000 |
| SECONDARY ANTIBODIES | | |
| HRP Goat Anti-Mouse Ig | *BD Pharmingen* | 1:500 – 1:10000 |
| Anti-rabbit IgG, HRP linked Antibody | *Cell Signaling Technology* | 1:5000 – 1:10000 |

**Supplementary Methods**

## Peripheral blood mononuclear cell (PBMC) isolation

A small sample of peripheral blood (5-10 mL) was collected from a healthy volunteer and a DMD patient carrying a mutation in the *DMD* gene (deletion of exons 48-50) (*Supplementary Table 1*) upon obtaining an informed consent in accordance with the Declaration of Helsinki and with the approval of the Institutional Review Board and the Bioethical Committee (number of approval: 122.6120.303.2016 and KB/111/2019) directly to the BD Vacutainer® CPT™ Mononuclear Cell Preparation Tube (*BD Biosciences*) containing Ficoll, sodium heparin and a gel plug. PBMC were isolated according to the manufacturer's instructions. Obtained pellet of PBMC was resuspended in PBMC medium containing complete StemPro medium (*Thermo Fisher Scientific*) supplemented with 2 mM L-Glutamine (*Thermo Fisher Scientific*) and the following cytokines: 100 ng/mL SCF, 20 ng/mL IL-3, 20 ng/mL IL-6 and 100 ng/mL FLT-3 (all from *Peprotech*).

## Reprogramming of PBMC to hiPSC

Reprogramming of PBMC was performed using Sendai vectors (*CytoTune™-iPS 2.0 Sendai Reprogramming Kit, Thermo Fisher Scientific*) according to the manufacturer’s protocol (feeder-free method). Subsequently, the characterization of the generated hiPSC lines was performed by staining for markers of pluripotency, differentiation of hiPSC *via* EB into cells originating from the three germ layers, and alkaline phosphatase (ALP) activity staining (described below).

## hiPSC culture

hiPSC were cultured on Geltrex™-coated 12-well plate wells in Essential 8 (E8) medium (*Thermo Fisher Scientific*), which was refreshed every day. When hiPSC colonies reached 80% confluence, cells were passaged using 0.5 mM EDTA and 10 µM Rho-associated protein kinase (ROCK) inhibitor Y-27632 (*Abcam*) was added to the medium for the first 24 hours after each passage. Cell culture was performed under standard conditions (37˚C, 5% CO_2_, 20% O_2_).

## CRISPR/Cas9-mediated generation of isogenic human induced pluripotent stem cells

The isogenic hiPSC lines were generated using CRISPR/Cas9 gene editing tool through introduction of mutation in the *DMD* gene (deletion of exon 50) in control DMB01 and DMB02 hiPSC derived from healthy donors (*Fig. 1a*) as described earlier^5^ and repair of the mutation in the *DMD* gene (deletion of exons 48-50) in hiPSC derived from the DMD patient (*Fig. 1b*).

In order to correct the mutation in the *DMD* gene, the sgRNA sequences were designed using the CHOPCHOP software (*Supplementary* *Table 2*) and cloned into the pSpCas9(BB)-2A-Puro plasmid (pSpCas9(BB)-2A-Puro (PX459) V2.0 was a gift from Feng Zhang (Addgene plasmid #62988; http://n2t.net/addgene:62988; RRID:Addgene_62988).^6^ Briefly, pSpCas9(BB)-2A-Puro plasmid was digested using the BpiI restriction enzyme (*Thermo Fisher Scientific*) and subsequently, it was subjected to electrophoretic separation on 1% agarose gel, followed by excision of the appropriate band from the gel and purification using the Zymoclean Gel DNA Recovery Kit (*Zymo Research*) according to the manufacturer’s instructions. Each pair of DNA oligos encoding the specificity-providing fragment of the sgRNA was mixed, phosphorylated using T4 PNK (*New England Biolabs*) and annealed to obtain oligoduplexes. The digested plasmid and annealed oligoduplexes were subjected to ligation reaction with the use of the Quick Ligation™ Kit (*New England Biolabs*) according to the vendor’s protocol. After ligation, the plasmid was used for the One Shot™ Stbl3™ Chemically Competent *E. coli* bacteria (*Thermo Fisher Scientific*) transformation, which was followed by plating on LB agar plates containing ampicillin and further plasmid amplification and isolation using the Plasmid MIDI AX (*A&A Biotechnology*) kit according to the manufacturer’s instructions.

In addition, another plasmid encoding repair template was constructed. In the first step, amplification of the sequences encoding the missing exons 48, 49 and 50 was performed using PCR. Subsequently, the sequences of the left and right homology arms were amplified by PCR. All products of amplification were subjected to electrophoresis on 2% agarose gel, and the proper bands were excised and purified using the Zymoclean Gel DNA Recovery Kit. The obtained sequences were linearly assembled with a use of overlap extension PCR. In the final step, both the pcDNA3.1-Hygro plasmid (*Thermo Fisher Scientific*) and the product of overlap extension PCR were digested with MluI and XhoI restriction enzymes (*New England Biolabs*) and ligated using the Quick Ligation™ Kit according to the manufacturer’s instruction. The One Shot™ Stbl3™ Chemically Competent *E. coli* bacteria were transformed with the obtained plasmid, which was followed by plating on LB agar plates containing ampicillin and further plasmid amplification and isolation using the Plasmid MIDI AX kit according to the manufacturer’s instructions.

## DNA isolation

DNA isolation was performed using the Genomic Mini kit (*A&A Biotechnology*) according to the manufacturer’s instructions. DNA concentration and purity were determined by the NanoDrop 1000 spectrophotometer.

## Nucleofection

The nucleofection of hiPSC was performed using the Human Stem Cell Nucleofector™ Kit 1 (*Lonza*) according to the manufacturer’s instructions. Briefly, 500,000 cells were suspended in 100 µL of the Human Stem Cell Nucleofector® Solution 1 and Supplement 1 mixture (at the 4.5:1 ratio). Subsequently, 5 µg of plasmid DNA was added and the mixture was transferred to a provided cuvette and subjected to nucleofection in the Amaxa Nucleofector (*Lonza*) using the A-023 program. Nucleofected cells were then seeded onto Geltrex™-coated wells in E8 medium supplemented with ROCK inhibitor. The next day, cells were selected by stimulation with 0.3 µM puromycin (*Sigma-Aldrich*) for 24 h and resistant cells were used in further procedures.

## Surveyor nuclease assay

In the first stage of the assay, the PCR reaction was prepared, consisting of 20 ng of DNA isolated from hiPSC after nucleofection (a mixture of cells or single clones) or control unedited hiPSC, filled up to 10 µL with water, 1.25 µL of each primer (sequences of primers used in this test are listed in *Supplementary Table 3*) and 12.5 µL KAPA2G Fast Genotyping Mix (*Sigma-Aldrich*) according to reaction conditions described in the manufacturer’s instructions.

Subsequently, 4.5 µL of PCR product from the control and tested samples were mixed with 1.5 µL of celery juice extract (CJE) buffer (prepared according to the protocol described by Till et al.^7^) and subjected to a heteroduplex formation reaction performed under the following conditions: 5 min at 95°C, temperature drop by 2°C/s to 85°C, followed by a temperature drop of 0.1°C/s to 25°C. The remaining volume of the PCR product was run on 2% agarose gel to confirm that the target sequence was amplified (data not shown). 1 μL of CJE containing mismatch-specific DNA endonuclease (prepared according to the protocol described by Till et al.^7^) and 3.5 μL of water were added to the product of the heteroduplex formation reaction. The samples were incubated for 45 min at 45°C and then the reaction product was analyzed on a 2% agarose gel.

## Screening of CRISPR/Cas9-modified single cell clones

Nucleofected hiPSC after preliminary validation of editing efficiency using Surveyor nuclease assay were seeded for single cell-derived clones by transfer of 700 cells to a 10 cm^2^ cell culture plate. After growing to the appropriate size, single cell clones were scratched and transferred to separate wells of the 96-well plate. Subsequently, DNA was isolated from each hiPSC clone and subjected to PCR genotyping using primers flanking upstream and downstream exon 50 (using the following primers – forward: 5’-CCCTTCAAACTTGTCCTGCTTTGAG-3’, reverse: 5’-TTGTCCTTCCACTCTCAGGCAGC-3’) and primers flanking upstream and downstream of the *DMD* exon 47 (using the following primers – forward: 5’-AAGGTAGTTGGAATTGTGCTG-3’, reverse: 5’-CTTGTGGCGAACGTTAGTGTAC-3’), for DMB02 and DMB03 hiPSC clones, respectively.

*Differentiation of hiPSC via EB*

3,000 hiPSC were seeded into non-adherent U-shaped 96-well plate in Essential 6™ (E6) medium (*Thermo Fisher Scientific*) containing 4 mg/mL polyvinyl alcohol (*Sigma-Aldrich*) and 10 µM Y-27632 ROCK inhibitor. After three days, the created EB were transferred to Geltrex™-coated 48-well plate and subjected to spontaneous differentiation for two weeks with E6 medium refreshed every other day. Eventually, the cells were fixed and stained for markers of all three germ layers-derived cells: vimentin, alpha smooth muscle actin (αSMA), GATA Binding Protein 4 (GATA-4) and neurofilament heavy chain (NFH).

## Immunofluorescent staining

Cells were fixed with 4% paraformaldehyde (PFA) (*Santa Cruz Biotechnology*) for 15 minutes at room temperature (RTemp) and washed with PBS. Afterwards, 0.1% Triton-X-100 (*BioShop Canada Inc.*) was added for permeabilization (15 min, RTemp) and washed again with PBS. In order to block non-specific binding sites, cells were incubated for 1h at RTemp with 3% bovine serum albumin (BSA) in PBS (blocking buffer). Primary antibodies (listed in *Supplementary Table 4*) diluted in blocking buffer were added for overnight incubation at 4°C. Next day, cells were washed three times with PBS and secondary antibodies (*Supplementary Table 4*) diluted in PBS were added for 1h at RTemp. After that, the cells were washed again three times with PBS and the nuclei were counterstained with 0.2 µg/mL 4',6-diamidyno-2-fenyloindol (DAPI) (*Sigma-Aldrich*). Actin filaments were stained using Phalloidin-iFluor 647 Reagent (*Abcam*) according to the manufacturer’s instruction. The images were captured using the Nikon Eclipse TS100 fluorescent microscope or the Carl Zeiss LSM-510 meta laser scanning confocal microscope.

*ALP activity staining*

For the ALP staining, hiPSC were seeded onto Geltrex™-coated wells of a 48-well plate and after two days they were subjected to ALP activity staining using the Leukocyte Alkaline Phosphatase Kit (*Sigma-Aldrich*) according to the manufacturer’s instructions.

## Karyotyping

Karyotype analyzes were performed by the Kariogen laboratory (Kraków, Poland) using the G-banding method.

## Cardiac differentiation

hiPSC were differentiated to cardiomyocytes according to the protocol described by Lian et al.^8^ Briefly, 3×10^4^ hiPSC were seeded into wells of a 24-well plate and cultured in E8 medium until they reached 90% confluency. At that time (day 0), cells were stimulated for 24 hours with CHIR99021 (*Sigma-Aldrich*) in the concentration range of 8-12 µM (cell line-dependent) in RPMI 1640 medium (*Biowest*) complemented with B-27 Supplement Minus Insulin (*Thermo Fisher Scientific*). On day 3, cells were stimulated with 5-7 µM (cell line-dependent) IWR-1 (*Sigma-Aldrich*) for two days and refreshed with RPMI 1640 medium with B-27 Supplement Minus Insulin. From day 7, differentiating cells were cultured in RPMI-1640 medium supplemented with B-27 (*Thermo Fisher Scientific*). To increase the purity of the cardiomyocyte population and the differentiation yield, differentiating cells were cultured from day 10^th^ to day 16^th^ in RPMI glucose-depleted medium (*Thermo Fisher Scientific*) supplemented with 4 mM sodium DL-lactate (*Sigma-Aldrich*). After that, cells were detached using TrypLE™ Select Enzyme (*Thermo Fisher Scientific*), harvested in RPMI 1640 medium supplemented with 20% fetal bovine serum (FBS) (*Biowest*), centrifuged at 200 × g for 5 minutes and reseeded onto Geltrex™-coated wells in RPMI medium supplemented with B-27.

## Evaluation of cardiac differentiation efficiency

The efficiency of cardiac differentiation was assessed to optimize the concentrations of small molecules used in the differentiation protocol, as well as to confirm the purity of the hiPSC-CM population intended for transcriptomic and proteomic analyses. The assay was performed using flow cytometry, on hiPSC-CM, which completed the entire 21-day protocol of differentiation. Cells were dissociated with TrypLE™ Select Enzyme and harvested in RPMI 1640 medium supplemented with 20% FBS into round-bottom polystyrene test tubes. All steps from that point were performed on ice. After centrifugation (250 × g, 5 min), cells were fixed in 4% PFA for 15 min and permeabilized using 0.1% Triton-X-100 in PBS for 20 minutes. Subsequently, cells were washed with PBS supplemented with 2% FBS (PBS + 2% FBS) and centrifuged (300 × g, 10 min). Primary anti-troponin T antibodies (Cardiac Troponin T Monoclonal Antibody (13-11), *Thermo Fisher Scientific*) were diluted in a ratio of 1:1000 in PBS + 2% FBS and added to the cells for 45 min incubation. Following this step, hiPSC-CM were washed with PBS + 2% FBS, centrifuged (300 × g, 5 min) and incubated with secondary antibodies AF488 Goat Anti-Mouse IgG (*Thermo Fisher Scientific*) diluted 1:500 in PBS + 2% FBS for 20 minutes in darkness. Finally, cells were washed again with PBS + 2% FBS and centrifuged (300 × g, 5 min). The pellet was suspended in 300 µL of PBS and 0.2 µg/mL DAPI was added. The analysis was performed using the LSR Fortessa cytometer (*BD*).

*Micro-electrode array (MEA) analysis*

To evaluate the electrophysiological activity of control and DMD hiPSC-CM, cells were seeded on Geltrex™-coated MEA 24-well plate (*Multi Channel Systems*) in 0.5 ml RPMI-1640 medium supplemented with B-27. After 24 h fresh 0.5 mL of medium was added and the cells were further cultured for 3 days. Subsequently, spontaneous electrophysiological activity of hiPSC-CM was recorded using Multiwell-MEA-System (*Multi Channel Systems*) and the Multiwell-Screen software (*Multi Channel Systems*) and the cells were stimulated with either 20 µM deferoxamine dissolved in water or 10 µM pioglitazone dissolved in DMSO. For that purpose, 200 µL of old medium was removed and the same volume of fresh medium with 5 times concentrated compounds was added to the cells. hiPSC-CM treated with either culture medium without any compound or corresponding volume of DMSO served as a control. Electrophysiological activity was then measured 24 h, 48 h and 72 h after stimulation. Analysis of obtained signals was performed using Multiwell-Analyzer software (*Multi Channel Systems*). Data are presented as the distribution of signals measured from all active electrodes.

## Protein isolation

Cells were washed twice with PBS without ions and then lysed for 20 min in 1% Triton-X-100 (*BioShop Canada Inc.*) in PBS supplemented with cOmplete™ Protease Inhibitor Cocktail (*Sigma-Aldrich*) on ice. Subsequently, cells were centrifuged at 14,000 × g for 20 min, 4°C and the supernatants were collected in separate tubes. For proteomic analyzes, both hiPSC and hiPSC-CM were lysed in 10% of sodium dodecyl sulfate (SDS) (*BioShop Canada Inc.*) in PBS instead of Triton-X-100. Protein concentration was determined using a bicinchoninic acid assay (*Sigma-Aldrich*) according to the manufacturer’s instructions.

*RNA isolation*

RNA was isolated according to the Chomczynski and Sacchi method^9^ using Fenozol (*A&A Biotechnology*). RNA concentration and purity were determined using the NanoDrop 1000 Spectrophotometer (*Thermo Fisher Scientific*).

## Transcriptome analysis (RNA Sequencing)

Before collecting the material, the high cardiac differentiation efficiency (above 80%) was confirmed by flow cytometric analyzes for each sample. RNA from control and DMD hiPSC-CM was isolated using Fenozol and the quality of the RNA was examined using the RNA 6000 Nano Kit (*Agilent*) on Agilent 2100 Bioanalyzer (*Agilent*). RNA libraries for a total of eight samples were prepared using the Ion AmpliSeq™ Transcriptome Human Gene Expression Kit (*Thermo Fisher Scientific*) according to the protocol provided by the manufacturer. Samples containing 60 ng of RNA were used as starting material. The libraries were then combined in equimolar amounts and sequenced on the Ion Proton™ Sequencer (*Thermo Fisher Scientific*) using the Ion PI™ Hi-Q™ Sequencing 200 Kit (*Thermo Fisher Scientific*) and the Ion PI™ Chip Kit v3 (*Thermo Fisher Scientific*).

## Proteome analysis

Before collecting the material, the high cardiac differentiation efficiency (above 80%) was confirmed by flow cytometry analyzes for each sample. Proteomic analysis was performed on a Q-Exactive Plus instrument coupled to an EASY nLC 1000 UHPLC system (both from *Thermo Fisher Scientific*). The peptides were separated on a 50 cm column packed in-house with C18 material (50 µm) using a hydrophobic gradient composed of 0.1% formic acid (Buffer A) and 0.1% formic acid in 80% acetonitrile (Buffer B). Buffer B increased from 5 to 34% within 215 min and was then ramped up to 55% within 5 min. Thereafter, % of buffer B was ramped up to 90% within 5 min and kept for 5 min. The column was then re-equilibrated with 5% B for 10 min. Full MS scans were performed at a resolution of 70,000, an automated gain control (AGC) target of 3e6 and a maximum injection time (IT) of 20 ms. The Top10 most abundant precursors were selected for fragmentation scans as a resolution of 17,500, an AGC target of 5e5 and a maximum IT of 60 ms. The normalized collision energy was set to 28.

*Bioinformatic analysis of transcriptome analysis data*

Bioinformatic analysis was performed on Torrent Suite™ Software v5.12.1 (*Thermo Fisher Scientific*). The readings were aligned to hg19 AmpliSeq Transcriptome ERCC v1 reference database and counted by the Torrent Coverage Analysis Plugin program. The obtained results of gene expression were normalized and differential analysis was performed with the DESeq2 package available in the R software version 3.3.3.

Differential expression analysis and Gene Set Enrichment Analysis (GSEA) were performed in R (v.4.1.2) using DESeq2 (v.1.34.0) and clusterProfiler (v.4.2.2), respectively. Batch effects estimation and correction were performed using sva (v.3.42.0) and limma (v.3.50.1) R libraries. The results were visualized using the ggplot2 (v.3.3.5), pathview (v.1.34.0), and enrichplot (v.1.14.2) libraries. GSEA was prepared on a set of all analyzed genes sorted by Fold Change value.

*Bioinformatic analysis of proteome analysis data*

The raw MS data were analyzed using MaxQuant analysis software and the implemented Andromeda software (1.5.3.8). Proteins were identified using the human UniProt database with common contaminants with trypsin set as protease and a maximum of 2 missed cleavages. All other parameters were set to default. Methionine oxidation and N-terminal acetylation were set as variable modifications, and cysteine carbamidomethylation was considered a fixed modification. Protein quantification was performed using the implemented LFQ algorithm with match-between-runs enabled. Statistical analysis was performed within Perseus (1.5.5.3). Potential contaminants and reverse peptides were excluded and intensities were log2-transformed. Two-sided *t* tests were performed to identify differentially expressed proteins between experimental conditions. Graphical visualization was performed with Instant Clue.

*Reverse transcription (RT) and quantitative PCR (qRT-PCR)*

Reverse transcription was performed using RevertAid Reverse Transcriptase polymerase (*Thermo Fisher Scientific*) according to the manufacturer’s protocol using 500 ng of RNA. The mixture for qRT-PCR contained 7.5 µL of SYBR Green JumpStart Taq ReadyMix (*Sigma-Aldrich*), 0.75 µL of forward primers and 0.75 µL of reverse primers (working concentration of 0.5 µM), 3 µL of water and 3 µL of complementary DNA (cDNA) (diluted 5 times after reverse transcription). Reaction was performed in StepOne Plus Real-Time PCR (*Applied Biosystems*) as presented in *Supplementary Table 5.* The sequences of the primers used in this study are provided in *Supplementary* *Table 6*. The troponin T (*TNNT)* gene served as a housekeeping gene.

## Western blot

10-20 µg of protein in 20 µL of lysis buffer and 5 µL of loading buffer was separated on polyacrylamide gel (6-12% depending on the molecular weight of the protein of interest) by electrophoresis. Subsequently, the proteins were transferred onto nitrocellulose membranes (*Bio-Rad*) through overnight wet transfer carried out at 30 V. The membranes were then blocked in non-fat 5% milk in Tris-buffered saline (TBS) with 0.1% Tween 20 (*BioShop Canada Inc.*) (TBST) buffer (blocking buffer) for 1 hour at room temperature (RTemp) and incubated overnight at 4°C with primary antibodies (listed in *Supplementary Table 7*) diluted in blocking buffer. The next day, the membranes were washed four times for five minutes in TBST buffer and incubated for 1 hour, at RTemp, with horseradish peroxidase (HRP)-conjugated secondary antibodies (*Supplementary Table 7*) diluted in blocking buffer. Afterwards, the membranes were washed again four times for 5 minutes in TBST buffer and chemiluminescence was detected using Immobilon Western Chemiluminescent HRP Substrate (*Sigma-Aldrich*) and ChemiDoc Imaging System (*Bio-Rad*).

## Preparation of reagents used for stimulation of hiPSC-CM

For the experiment with ROS and mitochondrial ROS determination, hiPSC-CM were stimulated with 20 µM deferoxamine mesylate salt (DFO) (*Sigma-Aldrich*) for 2 h. Stock solutions of DFO was freshly prepared in water at a concentration of 30 mM. Moreover, cells were stimulated with 10 µM pioglitazone (*MedChemExpress*) for 24 h (corresponding volume of DMSO was added to control cells as a vehicle control). To evaluate the effect of the Hippo pathway inhibition on mitoNEET expression, cells were stimulated with 10 µM TRULI for 24 h (corresponding volume of DMSO was added to control cells as a vehicle control).

**References**

1. Stępniewski J, Tomczyk M, Andrysiak K, Kraszewska I, Martyniak A, Langrzyk A, Kulik K, Wiśniewska E, Jeż M, Florczyk-Soluch U, Polak K, Podkalicka P, Kachamakova-Trojanowska N, Józkowicz A, Jaźwa-Kusior A, Dulak J. Human induced pluripotent stem cell-derived cardiomyocytes, in contrast to adipose tissue-derived stromal cells, efficiently improve heart function in murine model of myocardial infarction. *Biomedicines* 2020;**8**:1–21.

2. Chang LC, Chiang SK, Chen SE, Yu YL, Chou RH, Chang WC. Heme oxygenase-1 mediates BAY 11–7085 induced ferroptosis. *Cancer Letters* 2018;**416**.

3. Wu J, Bao L, Zhang Z, Yi X. Nrf2 induces cisplatin resistance via suppressing the iron export related gene SLC40A1 in ovarian cancer cells. *Oncotarget* 2017;**8**:93502–93515.

4. Sikorska K, Romanowski T, Stalke P, Izycka Swieszewska E, Bielawski KP. Association of Hepcidin mRNA Expression With Hepatocyte Iron Accumulation and Effects of Antiviral Therapy in Chronic Hepatitis C Infection. *Hepat Mon* 2014;**14**:e21184.

5. Martyniak A, Andrysiak K, Motais B, Coste S, Podkalicka P, Ferdek P, Stępniewski J, Dulak J. Generation of microRNA-378a-deficient hiPSC as a novel tool to study its role in human cardiomyocytes. *J Mol Cell Cardiol* 2021;**160**:128–141.

6. Ran FA, Hsu PD, Wright J, Agarwala V, Scott DA, Zhang F. Genome engineering using the CRISPR-Cas9 system. *Nat Protoc* 2013;**8**:2281–2308.

7. Till BJ, Zerr T, Comai L, Henikoff S. A protocol for TILLING and Ecotilling in plants and animals. *Nature Protocols* 2006;**1**.

8. Lian X, Zhang J, Azarin SM, Zhu K, Hazeltine LB, Bao X, Hsiao C, Kamp TJ, Palecek SP. Directed cardiomyocyte differentiation from human pluripotent stem cells by modulating Wnt/β-catenin signaling under fully defined conditions. *Nat Protoc* 2013;**8**:162–175.

9. Chomczynski P, Sacchi N. Single-step method of RNA isolation by acid guanidinium thiocyanate-phenol-chloroform extraction. *Anal Biochem* 1987;**162**:156–159.
